# A Polysorbate Structural Atlas Curated from Drift Tube Ion Mobility-Mass Spectrometry Measurements

**DOI:** 10.1021/acs.analchem.6c02138

**Published:** 2026-06-03

**Authors:** Kyle E. Lira, Alexander D. Goodness, Jody C. May, John A. McLean

**Affiliations:** Department of Chemistry, Center for Innovative Technology, Vanderbilt Institute of Chemical Biology, Vanderbilt Institute for Integrative Biosystems Research and Education, Vanderbilt-Ingram Cancer Center, 5718 Vanderbilt University, Nashville, Tennessee 37235, United States

## Abstract

Polysorbates (PSs) are mixtures of synthetic surfactants with widespread use in different application areas. PSs are particularly important in pharmaceutical and biotherapeutic formulations owing to their ability to minimize aggregation and surface adsorption. However, PSs are known to undergo degradation and autoxidation which can alter their efficacy. While studies have been conducted to better understand the chemical composition of PSs, comprehensive chemical characterization is challenging due to the heterogeneity of commercial PSs which contain numerous molecules representing both active surfactants and byproducts of synthesis. These complex mixtures result in extensive isomer and isobar species confounding characterization. Here, we utilize structurally selective drift tube ion mobility-mass spectrometry (DTIMS-MS) to compile a collision cross section (CCS) structural atlas for commonly found species in both polysorbate 20 and 80 (PS-20 and PS-80), two of the most commonly used PSs. This comprehensive IM-MS survey discovered a total of 536 molecules with discrete chemical formulas (350 in PS-20 and 186 species in PS-80), with each CCS measurement exhibiting high analytical reproducibility (<0.4% RSD, *n* ≥ 4 replicates). The primary chemical species found from electrospray spectra of US Pharmacopeia standards were singly charged monoesters and nonesterified species, with larger PS molecules favoring higher charge states. This first-of-a-kind, highly curated CCS database for PSs resulted in 41 empirically defined mobility-mass correlations (PS structure vs mass) that predict the location of individual PS oligomer species from IM-MS data for improved characterization.

## Graphical abstract

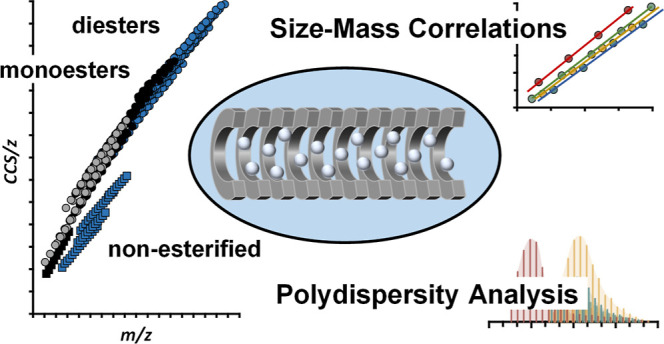

## Introduction

Polysorbates (PSs) are nonionic synthetic surfactants commonly utilized to improve solubility and bioavailability of compounds in a variety of commercial applications. While the specific mechanisms by which PSs improve solubility, emulsification, and surface adsorption are still areas of active research,^1–5^ it is generally understood that the amphiphilic nature of the PS chemical structure allows it to adhere to surfaces^6,7^ and to form micelles^8^ which promotes the preservation of constituents of interest, such as active pharmaceutical ingredients (APIs).^1,9^ For example, PSs are used in pharmaceuticals as excipients to help minimize protein aggregation and to stabilize the structural conformation of proteins to preserve their desired biological activity.^10^ Since PSs are thought to have low toxicity^11^ and high biocompatibility,^12^ they are also routinely included in cosmetics and food.^13,14^

The most commonly used PS mixtures are PS-20 and PS-80.^12^ Commercial PSs are prepared using sorbitol and a characteristic fatty acid (lauric acid for PS-20 and stearic acid for PS-80) in a multistep synthesis involving cyclization, esterification, and ethoxylation to produce polysorbate. Both PS-20 and PS-80 are distinguished by the desired end product, defined as a sorbitan headgroup functionalized with 20 ethylene oxide (EO) subunits and a single esterified laurate or oleate, respectively (Figure 1). However, commercial PS-20 and PS-80 are also comprised of a heterogeneous mixture of synthetic byproducts, unreacted precursors, and degradants, the latter of which has been an area of particular interest given its implications on product quality, protein stability, and pharmacokinetics.^15^

**Figure 1:**
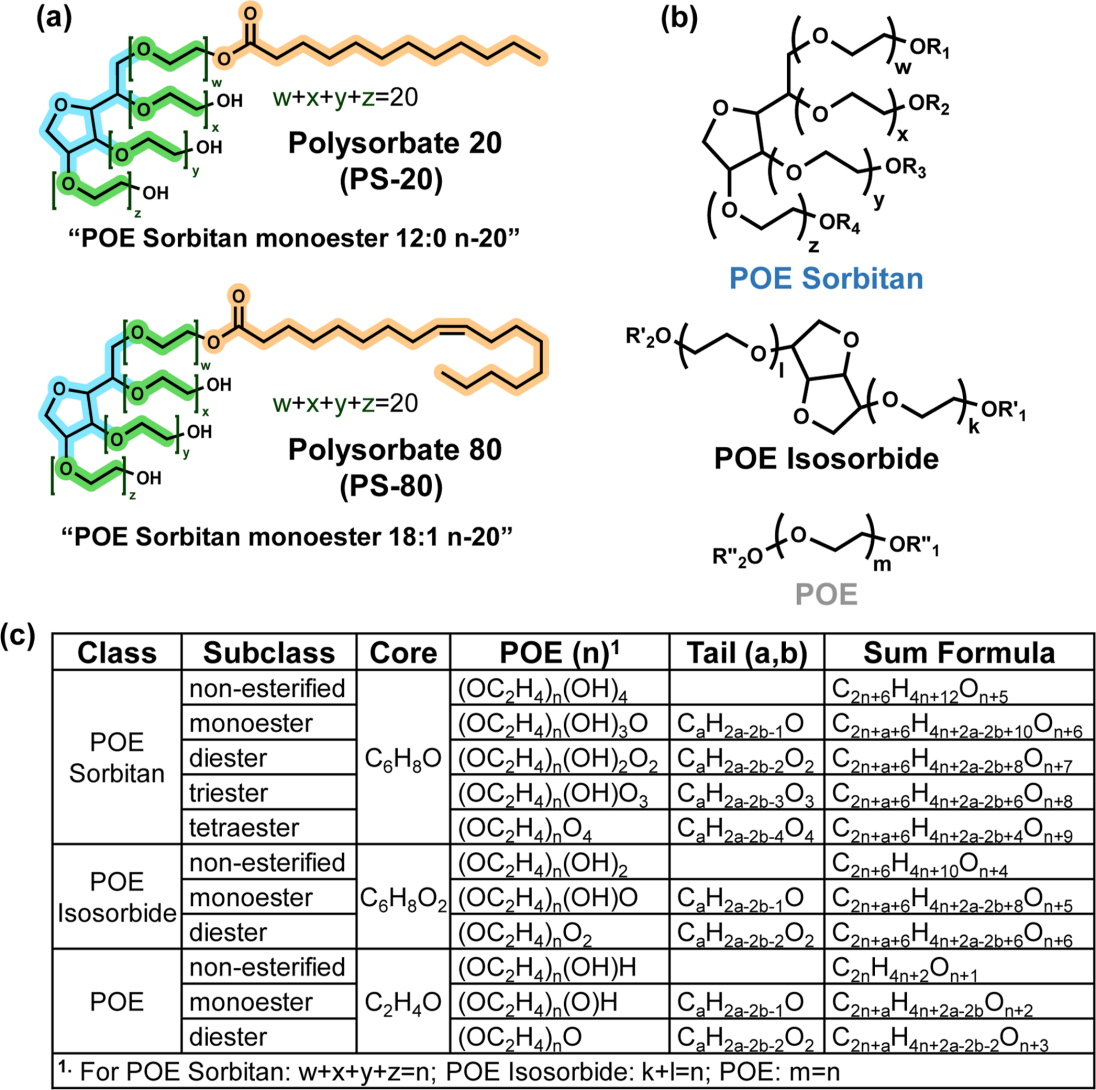
(a) Primary structural motifs for PS-20 and PS-80. Structures vary based on differences in the hydrophilic headgroup (blue), hydrophobic tails (gold), and/or repeating number of ethylene oxide subunits (green). For both PS-20 and PS-80, the primary structure involves a sorbitan headgroup functionalized at various positions with an average of 20 oligomers of ethylene oxide (i.e., polyoxyethylene or “POE”), with the esterification of a laurate or oleate, respectively. (b) General structure of the three series found in heterogeneous PS mixtures. Esterification is possible at each terminal site (R), otherwise a hydroxyl group is found at the site. (c) Formula breakdown for each PS series used to develop a mass target list of theoretically possible PS species.

Though PSs are used routinely as stabilizers, they are also known to undergo degradation and auto oxidation accelerated by exposure to heat and light^12,16,17^ which, in turn, affects product stability and lead to cleaved byproducts with known adverse health effects.^18–20^ For example, enzymatic cleavage of ester bonds results in degradants with the propensity to disrupt micelle formation and/or promote undesirable aggregation of fatty acids within the body.^21,22^ This concern has led to a growing interest in the investigation of PS degradation and the potential risks posed by PS degradants and byproducts.

Since commercially synthesized PSs are highly heterogeneous mixtures comprised of numerous structurally similar species, they are analytically challenging to characterize. Consequently, many analytical methods have been used to meet specific needs related to PS characterization and quantification. These have included both indirect (colorimetric method^23^ and analysis of fatty acid composition^24^) and direct (NMR,^25^ MALDI-TOF,^26,27^ FTICR^28^) methods. Among these proposed methodologies, mass spectrometry (MS) has proven to be a sensitive and selective option for characterizing individual species within PS; specifically in combination with liquid chromatography (LC–MS).^29–32^ Nevertheless, LC–MS is a relatively low-throughput technique (several minutes) and has a limited ability to differentiate isomeric species, thus necessitating additional separation dimensions exhibiting selectivity for distinguishing subtle structural differences.

This has motivated the present studies to evaluate structurally selective ion mobility spectrometry (IM) and the use of IM-derived collision cross section (CCS) measurements to reproducibly characterize PSs. IM is a postionization gas-phase electrophoretic technique which rapidly (μs to ms) separates ions based on their size, shape, and charge.^33,34^ Conventional drift tube ion mobility spectrometry (DTIMS) utilizes a uniform electric field to separate analytes in a time dispersive manner.^35^ Since variables in a DTIMS experiment such as pressure, temperature, and voltage are constant and well controlled, a fundamental ion mobility equation derived from first-principles of the kinetic theory of gases can be utilized to obtain highly accurate CCS values from drift tube measurements.^36^

Here, we utilize DTIMS-MS to comprehensively characterize PS-20 and PS-80 on the basis of size/shape (or structure) and mass (or chemical formula). The body of literature to date does not report any experimentally derived CCS values for PS-20, while for PS-80 only calibrated CCS values obtained from traveling wave IM instrumentation (^TW^CCS_N2_) have been reported, and only for a subset of nonesterified impurities and byproducts present in PS-80.^37^ Thus, an initial motivation of this study was to utilize DTIMS-MS to obtain highly accurate ^DT^CCS_N2_ measurements for the majority of species present in both PS-20 and PS-80. PS species identifications tentatively identified by exact mass measurement are assigned higher confidence using complementary analytical information, including expected ion adducts, LC retention time, and chromatographic elution trends. The resulting ^DT^CCS_N2_ database is subsequently combined with mobility-mass correlation analysis to validate measurements and predict the location of new species that were not prioritized in the initial surveys, resulting in the development of a ^DT^CCS_N2_ structural atlas, containing both validated CCS measurements and empirical mobility-mass correlation intervals, that serves as a reference for future PS studies.

## Experimental Methods

### Materials

Both high purity (Croda International, Millipore Sigma) and US Pharmacopeia (USP, Millipore Sigma) standards of PS-20 and PS-80 were analyzed to characterize species present in different PS mixtures. Standards were received as neat liquids and prepared in both acetonitrile and methanol (Optima LC–MS grade, Fisher Scientific) at 10 μg/mL. Samples were handled in a fume hood in the dark and stored in the dark at 4 °C to minimize exposure to factors which may induce degradation. Five interday technical replicates of both PS-20 and PS-80 standards were acquired to assess the reproducibility of CCS measurements.

### Instrumentation and Data Acquisition

All PS samples were analyzed via electrospray ionization (ESI) in positive ionization mode using DTIMS-MS (6560 Ion Mobility-QTOF, Agilent Technologies) and introduced via either an autosampler (LC–MS, see below) or by direct infusion (10 μL/min) the latter using an acquisition ranging between 1 and 2 min. Prior to analysis, the mass and mobility dimensions were calibrated using a mixture of phosphazenes (ESI-L Tuning Mix, Agilent), with calibrants being used to obtain direct CCS measurements via the single field approach implemented in MassHunter IM-MS Browser (10.0, Agilent).^38^ Reference CCS values used for calibration and a full list of optimized parameters are provided in Table S1.

### HPLC-MS Analysis of PS-20

LC-IM-MS was used to provide additional peak capacity, reduce ion suppression, and allow for the assessment of relative retention time orderings for validation purposes. Specifically, a deep coverage, 46 min reversed-phase LC method incorporating two elution modes was developed for this work, with approximately half of the method operating in a high organic, isocratic elution mode to account for PS components having a high affinity for the RPLC column (Zorbax Eclipse Plus C-18, 2.1 × 100 mm, 1.8 μm, Agilent). LC solvents were H_2_O (w/0.1% FA) and ACN (w/0.1% FA) as polar and nonpolar mobile phases. The LC gradient and resulting example chromatograms are provided in Figure S1.

### PS Identification and Nomenclature

An in silico database consisting of 37,080 mass targets for most theoretically possible PS species was developed (Supporting Information Excel Sheet, ES1), which accounts for both sodium and potassium ion adducts, and multiply charged species (*z* ≥ 2). Deisotoped IM-MS features were manually extracted as peak centroids using MassHunter IM-MS Browser and identifications were evaluated based on mass accuracy, LC elution ordering, and oligomer distributions mapped in CCS space via mobility-mass correlation prediction and confirmation.^39^ For empirical mobility-mass correlation fits, a minimum of three CCS-mass features corresponding to each PS structural series were fitted to a linear equation which were subsequently used to iteratively locate and validate additional molecular features in the series. Nomenclature for identified PS species specifies the headgroup/series, number of functionalized ethylene oxide units, degree of esterification, and the number of carbons and double bonds found in the fatty acid tails.^40^

### Photoirradiation Analysis

To induce photooxidation, a previously constructed custom-built device supporting a low-pressure mercury lamp (81-1057-51, BHK Inc.) was suspended over open vials containing samples, exposing samples to emission bands spanning from 185 to 579 nm (Figure S2).^41^ PS standards and blanks were irradiated for various time intervals (5, 10, 15 min) and compared to nonexposed USP standards. Analysis focused on signal intensity changes across different PS classes and subclasses, with specific attention focused on species prone to light degradation.

## Results and Discussion

### Solvent Evaluation

PS-20 and PS-80 standards (Croda) were analyzed in both acetonitrile and methanol, with particular focus on ionization efficiency and differences in oligomer distributions. While slight signal improvements were observed for PS standards prepared in methanol, both solvents demonstrated reproducibility in the mass and mobility dimensions which enabled subsequent workflows to utilize either solvent.

### Identification of Polysorbate Features and Structural Series

Using the targeted in silico mass database, identifications of PS compounds were made from DTIMS-MS data on the basis of accurate mass measurement (±10 ppm) and expected isotope ratios, which were subsequently validated using both the expected elution ordering from liquid chromatography and regression analysis from mobility-mass correlations. A total of five interday replicates were acquired (three from Croda, two from USP), and only compound features observed in four replicates were included in statistical analyses. A full list of identifications is summarized in Tables S2 and S3. Repeating patterns of features in the data were evident in the 2-dimensional CCS-mass conformational space plots and were found to correlate with structural series, number of fatty acids, charge carrier type, and charge state (Figure 2). These mobility-mass correlations represent structurally homologous series of PS compounds which are used both to confirm nearby assignments and predict where additional signals are expected to reside in IM-MS conformational space. The majority of ion species observed were sodium adducts ([M + Na]^+^), although nonesterified compounds also exhibited a lower abundance series of singly charged potassium adducts ([M + K]^+^). All identified multiply charged species were doubly charged and exclusively coordinated with two sodium ions—higher-order charge state ions incorporating potassium (e.g., [M + 2 K]^+^ or [M + Na + K]^+^) were not observed. Mass accuracies for both PS-20 and PS-80 identifications typically were better than ±5.0 ppm; poorer mass accuracies (>5.0 ppm), when observed, were strongly correlated to low or high ion abundance and/or isotopic distribution interferences, all of which distort the mass peak and reduce peak centroid measurement accuracy.

**Figure 2:**
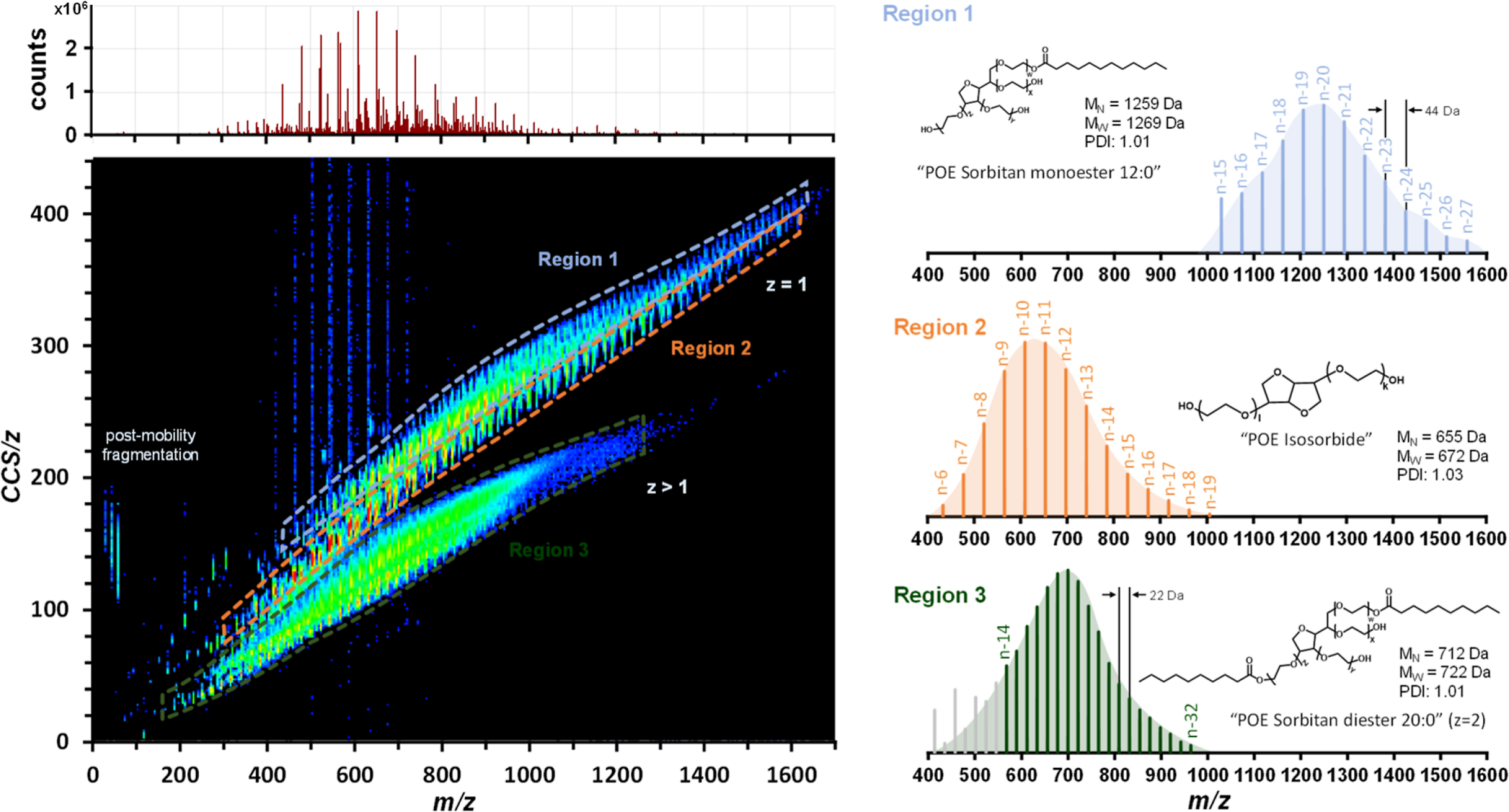
Visualization of a 2D IM-MS spectrum of PS-20. Using MS alone (*m*/*z*, *x*-axis), species can be identified to the chemical formula level; however, further structural annotation is challenging due to a convoluted spectrum containing numerous isomers that may exist under a single mass peak. With the addition of IM (CCS/*z*, *y*-axis), oligomer trends based on degree of esterification can be viewed in distinct regions of the heat map to provide additional confidence in identifications.

Analysis of the PS standards was also performed in negative ionization mode to determine if any additional structural information could be obtained. Species observed in negative ion mode consisted of almost exclusively deprotonated free fatty acids, ranging from lauric (12:0) to linoleic (18:2) acid (Figure S3). Abundance of free fatty acids was inversely proportional to their esterified counterparts, suggesting that these species are unreacted byproducts that did not esterify to the polysorbate structure during the initial synthesis.

### Validation of Identifications Using LC–MS

From the LC-IM-MS data sets, selected regions of the total ion chromatogram (TIC) where different PS species can be found are indicated in Figure S1. As expected, unbound PS species were the first to elute from the reversed-phase column given their hydrophilic properties and smaller size, followed by the monoester species with an elution order that corresponded to fatty acid chain length and hydrophilic headgroup. Larger PS species, such as polyoxyethylated (POE) sorbitan diesters, demonstrated a high affinity for the reversed-phase column and therefore eluted at 100% organic phase solvent composition during the extensive isocratic portion in the method. These observed elution orderings were highly consistent with previously reported LC–MS results^29,30,42,43^ and were used to validate the identifications of PS species.

### Curation of a CCS Database for PS-20 and PS-80

The specificity and reproducibility of IM measurements across different laboratories has demonstrated CCS values to be suitable chemical descriptors for increasing confidence in IM-MS-based identifications.^38^ This has prompted the development of searchable CCS databases which are specific to molecular classes such as lipids,^44^ peptides,^45^ and small molecule metabolites.^46^ The Unified CCS Compendium is one such resource, hosting a highly curated, self-consistent CCS repository of DTIMS measurements, with over 4000 experimentally derived CCS values covering 14 structurally based chemical super classes.^47^ For polysorbates, however, there is a widespread lack of CCS values currently available to aid in characterization and identification of PS species. One study obtained calibrated CCS values for PS-80 from TWIMS measurements, although only nonesterified species were reported (i.e., 226 CCSs are reported for various adducts with POE Sorbitan, POE Isosorbide, and POE with no fatty acids attached).^37^ To date, no experimentally measured CCS values have been reported for PS-20 species. The DTIMS-MS survey undertaken in this work yielded a large number of ^DT^CCS_N2_ values for PS-20 (362) and PS-80 (189), including both monoester and diester species found in the two PS standard mixtures investigated (Figure 3a). As illustrated in Figure 3b, all of the CCS values measured for PS-20 and PS-80 demonstrated a high degree of reproducibility (interday average % RSD <0.23%), which is significantly lower than the reported reproducibility threshold for ^DT^CCS_N2_ values of less than 0.5% RSD.^48^ Further, all PS CCS values curated in this survey are within the inclusion threshold for the Unified CCS Compendium (≤0.7% RSD).^47^ These highly curated CCS values are thus suitable as reference values to support the IM analysis of PSs and can serve as additional molecular descriptors and/or database algorithm parameters for integration into existing informatics solutions such as PolyMatch.^49^

**Figure 3:**
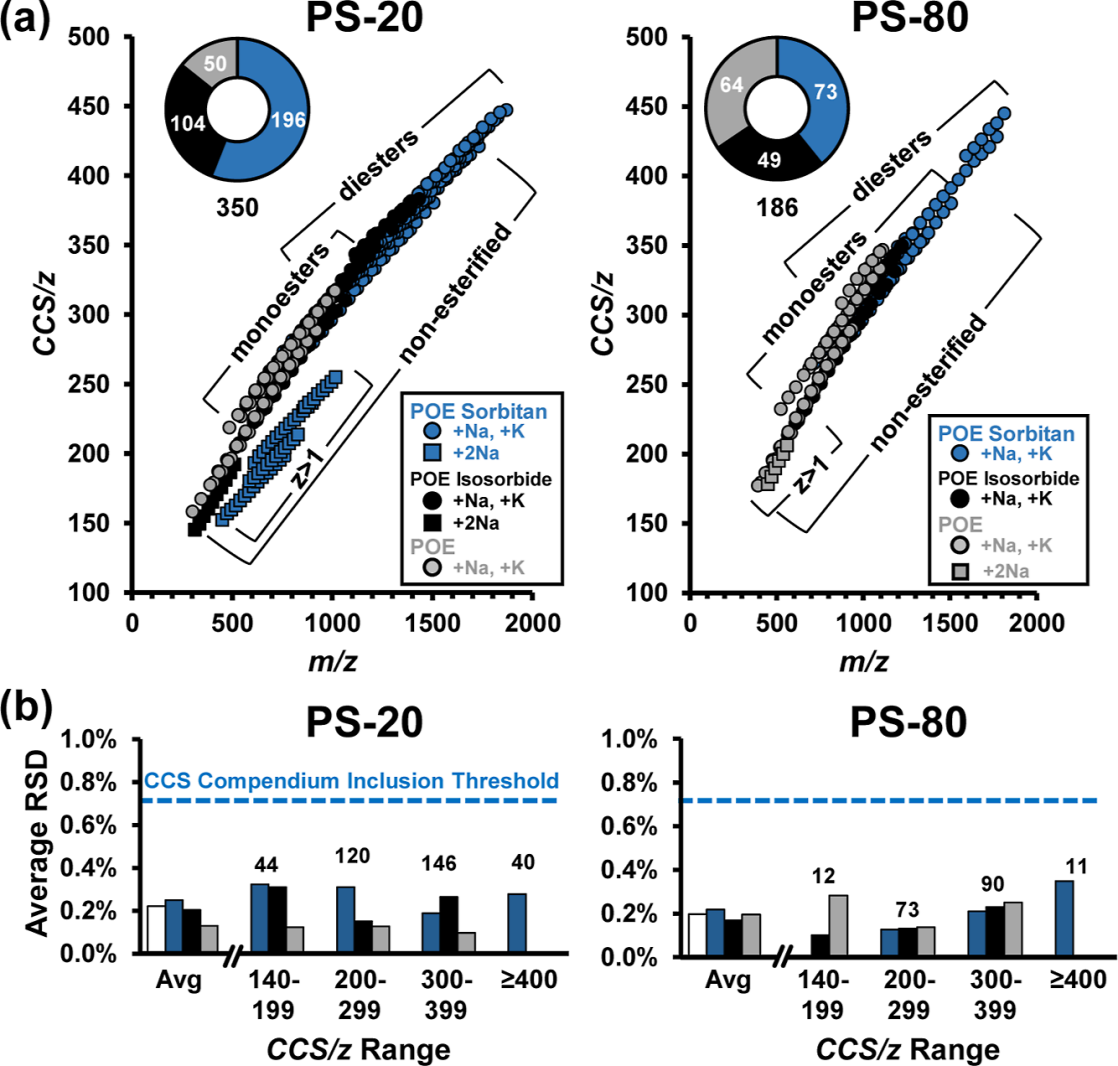
(a) CCS conformational space plot of all identified species within PS-20 and PS-80 standards comprised of four or more replicates. Specific oligomer distributions for the three different PS series are indicated, as well as varying charge states observed. While many species have similar or identical *m*/*z*, 2D IM-MS analysis allows for easy visualization of oligomer distributions. (b) Comparison of %RSD across all measurements (Avg) and within different *CCS/z* ranges. Numbers above the bars indicate the total number of species in each *CCS/z* range.

Among singly charged features, nonesterified species tend to occupy the lowest region of IM-MS space, with monoesters and diesters occupying higher regions of conformational space (Figure 3a). Main contributions to higher RSD are abundance (both low and high) and species found in the multiply charged region (Figure 3b). For example, the majority of identified sorbitan species in PS-20 were singly charged (73%) and had an average RSD of 0.21% compared to the multiply charged sorbitan species (27%) having an average RSD of 0.34%. In PS-80, all singly charged sorbitan species exhibit a CCS >200 Å^2^ with an average RSD of 0.22%.

### Empirical Regression Analysis of Polysorbate Structural Trends

Further investigation of the mobility-mass correlations observed in IM-MS conformational space reveals reproducible structural trends for the different PS species. Linear fits and confidence intervals (±0.4%) were applied to various PS structural series such as the POE Sorbitan monoester series (8:0 to 18:1) (Figure 4a), adding confidence to putative identifications based on exact mass, especially those of lower abundance or poor resolution. A similar dual–series correlation strategy was also previously utilized for compiling a structural atlas of lipids, where two sets of linear fits corresponded to acyl chain carbon numbers and degrees of unsaturation.^44^ Whereas both linear and power law equations exhibited a high degree of correlation (*R* ^2^ ≥ 0.994) to these observed structural trends (Table S4), linear fits are easier to implement and exhibit less extrapolation error than higher-order fits. Thus, linear mobility-mass correlation equations were generated for all observed PS structural trends (25 for PS-20 and 16 for PS-80) and these 41 equations were subsequently used to validate the CCS database compiled in this work, and to predict the location of features corresponding to PS species that were not initially extracted from the IM-MS data sets (Figure 4b). More specifically, these mobility-mass correlations were subsequently used to identify an additional 122 species in PS-20 and 88 species in PS-80 that were masked in the initial surveys.

**Figure 4:**
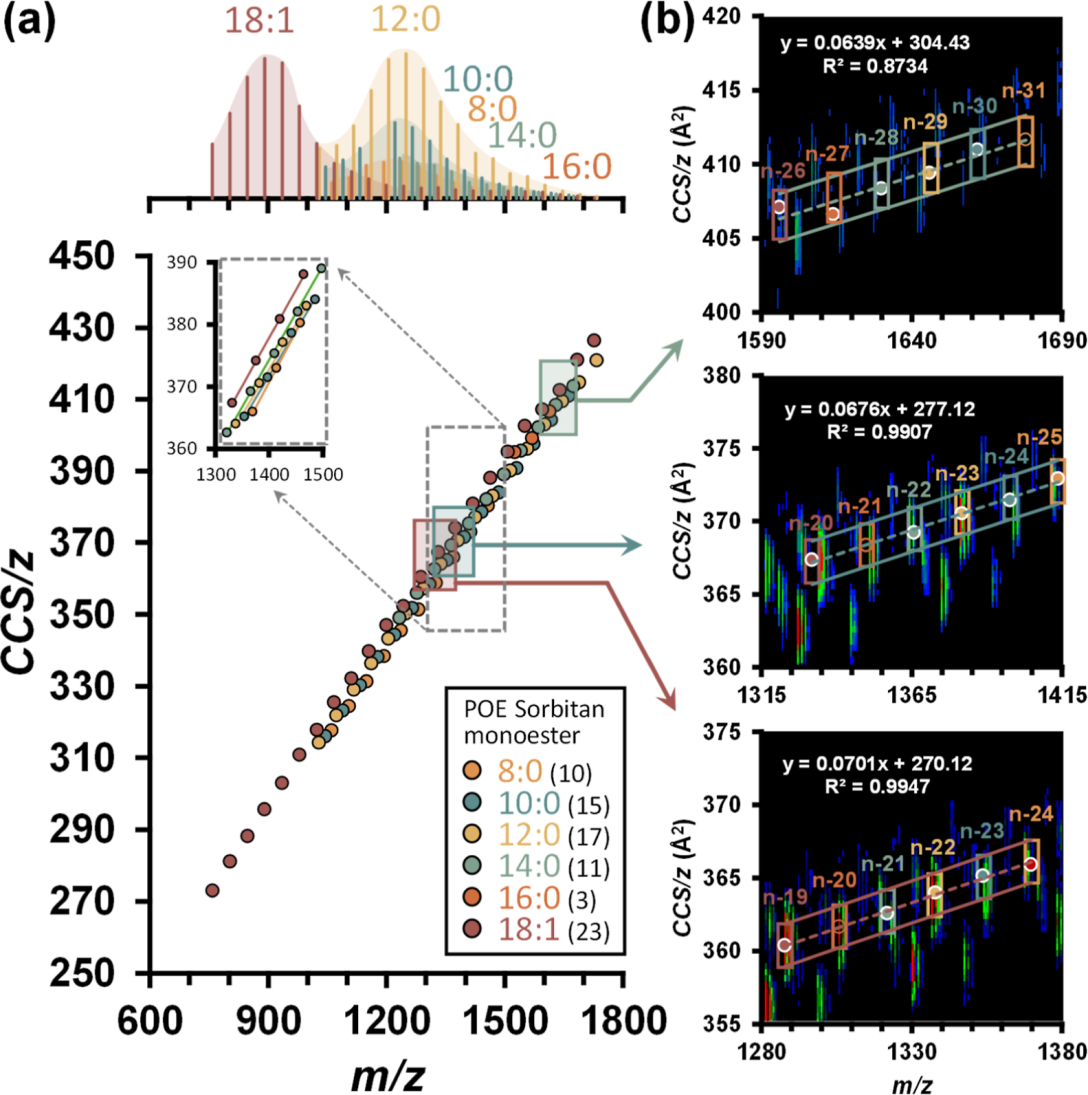
(a) Trendline analysis of PS Sorbitan series in IM-MS conformational space. Degree of esterification and ethoxylation both follow linear trends and can be used to add confidence to neighboring PS species of a different series. (b) Select examples of linear trends across different PS structural series, spanning multiple mass ranges. Fit equations were created and used qualitatively to identify and/or confirm PS species of low abundance or resolution. In the top box, the fit equation and confidence intervals were used to predict where a specific PS species (POE Sorbitan monoester 8:0 *n*-31) would be found. The middle and bottom boxes demonstrated how features could be identified with confidence using surrounding identifications and fit equations, as well as how trends are consistently observed. A full list of trendline analysis details for select PS series are summarized in Table S3.

While the primary PS species were identified from the IM-MS measurements, these data sets still contain many unknown features which have yet to be identified (Figure S4). One of the contributing factors confounding complete identification of all observed signals is the transient nature of many of the lower abundance features which, for reasons of ionization efficiency and ion suppression, do not appear in all replicates. Additionally, for the ESI analysis conducted in this study, larger PS species tend to shift the observed ion channels from singly-to multiply charged species, as the larger oligomer sizes have more surface area to accommodate multiple charge carriers. This results in multication species (+2Na, + 2K, + NaK, etc.) populating the higher charge state regions, which contributes to a high degree of complexity that complicates interpretation even with the addition of structurally selective IM measurements. As this work primarily focuses on singly charged PS ion species, the presence of these multiply charged signals means that oligomer distributions reported here are inherently biased to lower average molecular weights.

### Application of the Structural Atlas to Photodegradation Studies

As described above, PSs are prone to degradation due to a variety of factors. Light exposure of PS, for example, is reported to induce oxidation in PS and more susceptible in species with unsaturated fatty acids and POE chains.^31,50,51^ Thus, it is imperative to track potential photooxidation in PS mixtures, which could result in decreased stability and efficacy of pharmaceutical ingredients. Here, the utility of IM-MS conformational space was applied to simplify and add confidence to PS degradation analysis. By leveraging the CCS database and derived structural trends, the effect of photoirradiation on PS standards can be readily deconvoluted to visualize and quantify degradation (Figure 5a). Specifically, linear fits and confidence intervals were applied to the 3D IM-MS heat maps to isolate specific PS oligomer distributions of interest for monitoring the degree of degradation occurring in response to sample exposure to a high energy photon source (Figure 5b). Esterified PS species with an unsaturated fatty acid tail(s) (e.g., oleic acid) were chosen for this proof-of-concept study as these fatty acid esters are particularly susceptible to oxidation. Analysis of the POE Sorbitan monoester 18:1 series illustrates the impact of light exposure on PS samples, and the susceptibility to degradation under specific conditions (Figure 5c). An exposure time of 15 min results in an order of magnitude loss in signal for the complete POE Sorbitan monoester 18:1 series. At the same time, the nonesterified POE Sorbitan intensities increase nearly 2-fold after 15 min of exposure. Collectively, these observations indicate the acceleration of degradation pathways for PS species upon light exposure, as has been widely reported in the literature. The IM-MS structural atlas provides a basis for locating and identifying oligomers of interest in the spectra, and the corresponding CCS database and derived mobility-mass correlations described in this work may be readily integrated into quality assurance workflows.

**Figure 5:**
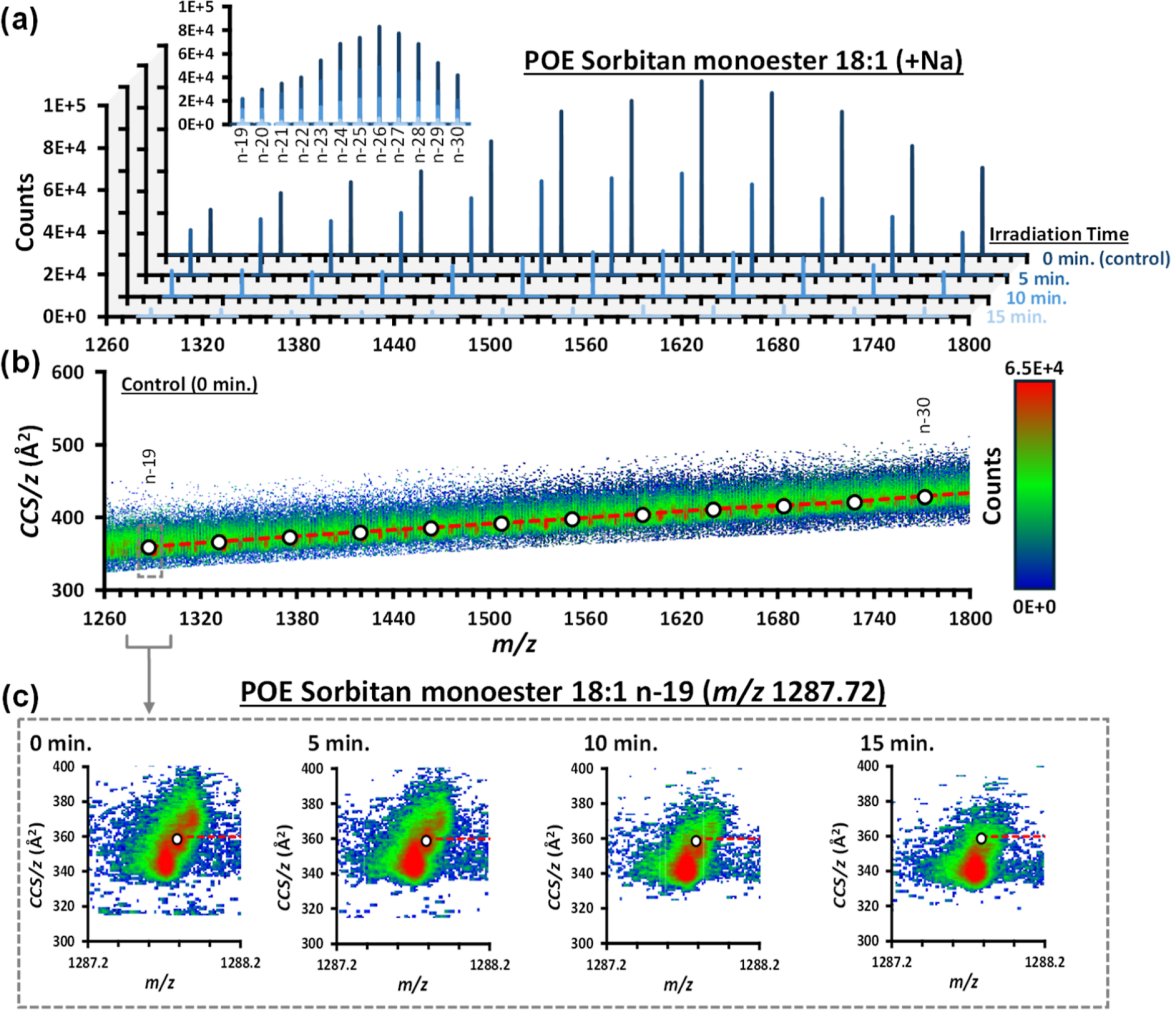
(a) Mass spectra for the POE Sorbitan monoester 18:1 series in PS-80 spanning from the monomers n-19 to n-30. (b) 3D IM-MS heat map of the same series prior to irradiation, with linear trendline and confidence interval (red dashes) overlaid to illustrate the utility of the fit equations in PS analysis. (c) Degradation of a specific monomer (POE Sorbitan monoester 18:1 n-19) after being irradiated with the mercury lamp over time intervals of 5, 10, and 15 min.

## Conclusion

Understanding the chemical composition of PS mixtures is of critical importance given its widespread use in a multitude of applications and products. This work presents the first experimentally derived ^DT^CCS database and empirical mobility-mass correlations (collectively referred to as a polysorbate structural atlas) for common species found in both polysorbate 20 and 80, and establishes a framework for utilizing this resource to support confident PS identifications and predict the location of specific PS species in IM-MS conformational space. Using structurally selective IM coupled with MS, the complexity of PS is revealed in high chemical detail with several hundred distinct chemical species found in standard mixtures of PS-20 and PS-80. Importantly, this work provides a baseline for experimenters to conduct differential studies comparing, for example, global changes in the structure and abundance of various PS species in response to small molecule additives or degradation (oxidation, temperature, light, etc.). A proof-of-concept example is provided for the photodegradation of PS-80, where depletion of fatty acid esters and augmentation of downstream nonesterified species was tracked in response to UV light exposure, though we note that more rigorously controlled experimental parameters would be needed to derive quantitative information from this work.

While this study provides a foundation for structural mapping of PS mixtures, there are a multitude of unidentified species and likely a large amount of unresolved isomeric species. Future experiments such as energy-resolved IM-MS and high resolution ion mobility (HRIM) are expected to build upon this work to provide additional dimensions of analytical information for the characterization of PSs and potentially uncover previously unresolved isomers present in complex PS mixtures.

## Supplementary Material





## Notes

Experimental parameters for DTIMS-MS analysis, LC-IM-MS analysis results, photoirradiation experimental setup, summary of all mass and ion mobility measurements for PS-20, summary of all mass and ion mobility measurements for PS-80, negative mode summary of PS-20 and PS-80 (USP), details of linear mobility-mass correlation fits for PS-20 and PS-80, and IM feature analysis of PS species (PDF)
File of in silico database of PS mass targets (XLSX)

## References

[1] Katakam M., Bell L. N., Banga A. K. (1995). Effect of Surfactants on the Physical
Stability of Recombinant Human Growth Hormone. J. Pharm. Sci..

[2] Saha P., Kou J. H. (2000). Effect of Solubilizing Excipients on Permeation of
Poorly Water-Soluble Compounds across Caco-2 Cell Monolayers. Eur. J. Pharm. Biopharm..

[3] Trotta M., Gallarate M., Pattarino F., Morel S. (2001). Emulsions Containing
Partially Water-Miscible Solvents for the Preparation of Drug Nanosuspensions. J. Contr. Release.

[4] Strickley R. G., Lambert W. J. (2021). A Review of Formulations of Commercially
Available
Antibodies. J. Pharm. Sci..

[5] Maa Y.-F., Hsu C. C. (1998). Investigation on
Fouling Mechanisms for Recombinant
Human Growth Hormone Sterile Filtration. J.
Pharm. Sci..

[6] Randolph, T. W. ; Jones, L. S. Surfactant-Protein Interactions. In Rational Design of Stable Protein Formulations: Theory and Practice; Carpenter, J. F. , Manning, M. C. , Eds.; Springer US: Boston, MA, 2002; pp 159–175. 10.1007/978-1-4615-0557-0_7.

[7] Mahler H.-C., Huber F., Kishore R. S. K., Reindl J., Rückert P., Müller R. (2010). Adsorption Behavior of a Surfactant
and a Monoclonal
Antibody to Sterilizing-Grade Filters. J. Pharm.
Sci..

[8] Knoch H., Ulbrich M. H., Mittag J. J., Buske J., Garidel P., Heerklotz H. (2021). Complex Micellization
Behavior of the Polysorbates
Tween 20 and Tween 80. Mol. Pharmaceutics.

[9] Chang B. S., Kendrick B. S., Carpenter J. F. (1996). Surface-Induced
Denaturation of Proteins
during Freezing and Its Inhibition by Surfactants. J. Pharm. Sci..

[10] Singh S. M., Bandi S., Jones D. N. M., Mallela K. M. G. (2017). Effect of Polysorbate
20 and Polysorbate 80 on the Higher-Order Structure of a Monoclonal
Antibody and Its Fab and Fc Fragments Probed Using 2D Nuclear Magnetic
Resonance Spectroscopy. J. Pharm. Sci..

[11] Ha E., Wang W., Wang Y. J. (2002). Peroxide
Formation in Polysorbate
80 and Protein Stability. J. Pharm. Sci..

[12] Kerwin B. A. (2008). Polysorbates
20 and 80 Used in the Formulation of Protein Biotherapeutics: Structure
and Degradation Pathways. J. Pharm. Sci..

[13] Sattler S., Gollomp S., Curry A. (2023). A Narrative
Literature Review of
the Established Safety of Human Serum Albumin Use as a Stabilizer
in Aesthetic Botulinum Toxin Formulations Compared to Alternatives. Toxins.

[14] Thakur R. K., Villette C., Aubry J. M., Delaplace G. (2007). Spectrophotometric
Method Associated with Formulation Scans for Application of Hydrophilic–Lipophilic
Deviation Concept in Food Emulsions. Colloids
Surf. Physicochem. Eng. Asp..

[15] Larson N. R., Wei Y., Prajapati I., Chakraborty A., Peters B., Kalonia C., Hudak S., Choudhary S., Esfandiary R., Dhar P., Schöneich C., Middaugh C. R. (2020). Comparison of Polysorbate
80 Hydrolysis and Oxidation on the Aggregation of a Monoclonal Antibody. J. Pharm. Sci..

[16] Donbrow M., Azaz E., Pillersdorf A. (1978). Autoxidation
of Polysorbates. J. Pharm. Sci..

[17] Jaeger J., Sorensen K., Wolff S. P. (1994). Peroxide
Accumulation in Detergents. J. Biochem. Biophys.
Methods.

[18] Knepp V. M., Whatley J. L., Muchnik A., Calderwood T. S. (1996). Identification
of Antioxidants for Prevention of Peroxide-Mediated Oxidation of Recombinant
Human Ciliary Neurotrophic Factor and Recombinant Human Nerve Growth
Factor. PDA J. Pharm. Sci. Technol..

[19] Herman, A. C. ; Boone, T. C. ; Lu, H. S. Characterization, Formulation, and Stability of Neupogen® (Filgrastim), a Recombinant Human Granulocyte-Colony Stimulating Factor. In Formulation, Characterization, and Stability of Protein Drugs: Case Histories: Case Histories; Pearlman, R. , Wang, Y. J. , Eds.; Springer US: Boston, MA, 2002; pp 303–328. 10.1007/0-306-47452-2_7. 8914196

[20] Singh S. K., Mahler H.-C., Hartman C., Stark C. A. (2018). Are Injection Site
Reactions in Monoclonal Antibody Therapies Caused by Polysorbate Excipient
Degradants?. J. Pharm. Sci..

[21] Hall T., Sandefur S. L., Frye C. C., Tuley T. L., Huang L. (2016). Polysorbates
20 and 80 Degradation by Group XV Lysosomal Phospholipase A2 Isomer
X1 in Monoclonal Antibody Formulations. J. Pharm.
Sci..

[22] Dixit N., Salamat-Miller N., Salinas P. A., Taylor K. D., Basu S. K. (2016). Residual
Host Cell Protein Promotes Polysorbate 20 Degradation in a Sulfatase
Drug Product Leading to Free Fatty Acid Particles. J. Pharm. Sci..

[23] Khossravi M., Kao Y.-H., Mrsny R. J., Sweeney T. D. (2002). Analysis
Methods
of Polysorbate 20: A New Method to Assess the Stability of Polysorbate
20 and Established Methods That May Overlook Degraded Polysorbate
20. Pharm. Res..

[24] Ilko D., Braun A., Germershaus O., Meinel L., Holzgrabe U. (2015). Fatty Acid
Composition Analysis in Polysorbate 80 with High Performance Liquid
Chromatography Coupled to Charged Aerosol Detection. Eur. J. Pharm. Biopharm..

[25] Zhang Q., Wang A., Meng Y., Ning T., Yang H., Ding L., Xiao X., Li X. (2015). NMR Method
for Accurate
Quantification of Polysorbate 80 Copolymer Composition. Anal. Chem..

[26] Ayorinde F. O., Gelain S. V., Johnson J. H., Wan L. W. (2000). Analysis
of Some Commercial Polysorbate Formulations Using Matrix-Assisted
Laser Desorption/Ionization Time-of-Flight Mass Spectrometry. Rapid Commun. Mass Spectrom..

[27] Frison-Norrie S., Sporns P. (2001). Investigating the Molecular
Heterogeneity of Polysorbate
Emulsifiers by MALDI-TOF MS. J. Agric. Food
Chem..

[28] Perez
Hurtado P., Lam P. Y., Kilgour D., Bristow A., McBride E., O’Connor P. B. (2012). Use of High Resolution Mass Spectrometry
for Analysis of Polymeric Excipients in Drug Delivery Formulations. Anal. Chem..

[29] Hewitt D., Alvarez M., Robinson K., Ji J., Wang Y. J., Kao Y.-H., Zhang T. (2011). Mixed-Mode and Reversed-Phase Liquid
Chromatography–Tandem Mass Spectrometry Methodologies to Study
Composition and Base Hydrolysis of Polysorbate 20 and 80. J. Chromatogr. A.

[30] Borisov O. V., Ji J. A., Wang Y. J., Vega F., Ling V. T. (2011). Toward
Understanding Molecular Heterogeneity of Polysorbates by Application
of Liquid Chromatography–Mass Spectrometry with Computer-Aided
Data Analysis. Anal. Chem..

[31] Borisov O. V., Ji J. A., Wang Y. J. (2015). Oxidative Degradation of Polysorbate
Surfactants Studied by Liquid Chromatography–Mass Spectrometry. J. Pharm. Sci..

[32] Zhang R., Wang Y., Tan L., Zhang H. Y., Yang M. (2012). Analysis of
Polysorbate 80 and Its Related Compounds by RP-HPLC with ELSD and
MS Detection. J. Chromatogr. Sci..

[33] Cohen M. J., Karasek F. W. (1970). Plasma Chromatography^TM^ A New Dimension
for Gas Chromatography and Mass Spectrometry. J. Chromatogr. Sci..

[34] Carr, T. W. Plasma Chromatography; Plenum Press: New York, NY, 1984; p 259.

[35] May J. C., McLean J. A. (2015). Ion Mobility-Mass Spectrometry: Time-Dispersive
Instrumentation. Anal. Chem..

[36] May J. C., Morris C. B., McLean J. A. (2017). Ion Mobility Collision Cross Section
Compendium. Anal. Chem..

[37] Fiebig L., Laux R. (2016). A Collision Cross Section
and Exact Ion Mass Database of the Formulation
Constituents Polyethylene Glycol 400 and Polysorbate 80. Int. J. Ion Mobil. Spectrom..

[38] Stow S. M., Causon T. J., Zheng X., Kurulugama R. T., Mairinger T., May J. C., Rennie E. E., Baker E. S., Smith R. D., McLean J. A., Hann S., Fjeldsted J. C. (2017). An Interlaboratory
Evaluation of Drift Tube Ion Mobility–Mass Spectrometry Collision
Cross Section Measurements. Anal. Chem..

[39] Rose B. S., May J. C., Picache J. A., Codreanu S. G., Sherrod S. D., McLean J. A. (2022). Improving Confidence
in Lipidomic Annotations by Incorporating
Empirical Ion Mobility Regression Analysis and Chemical Class Prediction. Bioinformatics.

[40] Yang K., Hewarathna A., Geerlof-Vidavsky I., Rao V. A., Gryniewicz-Ruzicka C., Keire D. (2019). Screening of Polysorbate-80 Composition by High Resolution Mass Spectrometry
with Rapid H/D Exchange. Anal. Chem..

[41] Harris R. A., May J. C., Stinson C. A., Xia Y., McLean J. A. (2018). Determining
Double Bond Position in Lipids Using Online Ozonolysis Coupled to
Liquid Chromatography and Ion Mobility-Mass Spectrometry. Anal. Chem..

[42] Solak
Erdem N., Alawani N., Wesdemiotis C. (2014). Characterization
of Polysorbate 85, a Nonionic Surfactant, by Liquid Chromatography
vs. Ion Mobility Separation Coupled with Tandem Mass Spectrometry. Anal. Chim. Acta.

[43] Li Y., Hewitt D., Lentz Y. K., Ji J. A., Zhang T. Y., Zhang K. (2014). Characterization and
Stability Study of Polysorbate 20 in Therapeutic
Monoclonal Antibody Formulation by Multidimensional Ultrahigh-Performance
Liquid Chromatography–Charged Aerosol Detection–Mass
Spectrometry. Anal. Chem..

[44] Leaptrot K. L., May J. C., Dodds J. N., McLean J. A. (2019). Ion Mobility Conformational
Lipid Atlas for High Confidence Lipidomics. Nat. Commun..

[45] Fenn L. S., Kliman M., Mahsut A., Zhao S. R., McLean J. A. (2009). Characterizing
Ion Mobility-Mass Spectrometry Conformation Space for the Analysis
of Complex Biological Samples. Anal. Bioanal.
Chem..

[46] Paglia G., Williams J. P., Menikarachchi L., Thompson J. W., Tyldesley-Worster R., Halldórsson S., Rolfsson O., Moseley A., Grant D., Langridge J., Palsson B. O., Astarita G. (2014). Ion Mobility Derived
Collision Cross Sections to Support Metabolomics Applications. Anal. Chem..

[47] Picache J. A., Rose B. S., Balinski A., Leaptrot K. L., Sherrod S. D., May J. C., McLean J. A. (2019). Collision Cross Section Compendium
to Annotate and Predict Multi-Omic Compound Identities. Chem. Sci..

[48] Morris C. B., May J. C., Leaptrot K. L., McLean J. A. (2019). Evaluating Separation
Selectivity and Collision Cross Section Measurement Reproducibility
in Helium, Nitrogen, Argon, and Carbon Dioxide Drift Gases for Drift
Tube Ion Mobility–Mass Spectrometry. J. Am. Soc. Mass Spectrom..

[49] Koelmel J. P., Stelben P., Oranzi N., Kummer M., Godri D., Qi J., Rennie E. E., Lin E., Weil D., Godri Pollitt K. J., PolyMatch K. J. (2024). Novel Libraries,
Algorithms, and Visualizations for
Discovering Polymers and Chemical Series. J.
Am. Soc. Mass Spectrom..

[50] Wu Y., Richards D., Zhang Y., Zhang L., Dodd S. W., Schöneich C. (2025). Mechanisms of Polysorbate 80 Oxidation: Acetate and
Fe­(III) Mediated near UV and Visible Light Degradation Enhanced by
Free Fatty Acids. Eur. J. Pharm. Biopharm..

[51] Yao J., Dokuru D. K., Noestheden M., Park S. S., Kerwin B. A., Jona J., Ostovic D., Reid D. L. (2009). A Quantitative Kinetic
Study of Polysorbate Autoxidation: The Role of Unsaturated Fatty Acid
Ester Substituents. Pharm. Res..

