# Supplementary material for: A Polysorbate Structural Atlas Curated from Drift Tube Ion Mobility-Mass Spectrometry Measurements
Source: Anal Chem. 2026 Jun 3;98(23):17331–9. doi: 10.1021/acs.analchem.6c02138 (PMC13276851; doi:10.1021/acs.analchem.6c02138)
Supplement: Supplementary file 1 [file ac6c02138_si_001.pdf]

Supporting Information

A Polysorbate Structural Atlas Curated From Drift Tube Ion Mobility-Mass Spectrometry Measurements

Kyle E. Lira, Alexander D. Goodness, Jody C. May, and John A. McLean*

Department of Chemistry, Center for Innovative Technology, Vanderbilt Institute of Chemical Biology, Vanderbilt-Ingram Cancer Center, and Vanderbilt Institute for Integrated Biosystems Research and Education, Vanderbilt University, Nashville, TN 37235-1822 (USA)

*Corresponding author email: john.a.mclean@vanderbilt.edu

Table of Contents

Table S1 Reference CCS Values and Parameters for DTIMS-MS…………… S2

Figure S1 LC-IM-MS Analysis Results………………………….………………….. S3

Figure S2 Photoirradiation Experimental Setup……………….…………………….S4

Table S2 Summary of All Mass and Mobility Measurements for PS-20……… S5-10

Table S3 Summary of All Mass and Mobility Measurements for PS-80……… S11-13

Figure S3 Negative Mode Summary of PS-20 and PS-80 (USP)……………… S14

Table S3 Details of Mobility-Mass Correlation Fits for PS-20 & PS-80……..... S15

Figure S4 IM Feature Analysis of PS Species ……………………………………S16

Table S1. Reference CCS Values and Experimental Parameters for DTIMS-MS.

m/z	Reference CCS Value (Å^2)
322	153.73
622	202.96
922	243.64
1222	282.20
1522	316.96
1822	351.25

CCS Calibrant Equation Used in this Work:

$$t_A = \frac{\beta}{z}\sqrt{\frac{mass_{ion}}{mass_{gas} + mass_{ion}}} \cdot CCS + t_{fix}$$

Agilent 6560 IM-QTOF with Dual AJS ESI Source — Positive Ion Mode

Source Parameters

Gas Temp	325°C
Drying Gas	5 L/min
Nebulizer	30 psi
Capillary Voltage	3800 V
Nozzle Voltage	2000 V
Sheath Gas Temp	275°C
Sheath Gas Flow	11 L/min

MS TOF

Fragmentor	400 V
Oct 1 RF Vp-p	750 V
Mass Range	*m/z* 20-3200
Trap Fill Time	70000 μs
Trap Release Time	200 μs
Frame Rate	1 Frame/s
Acquisition Mode	IM-QTOF, Positive

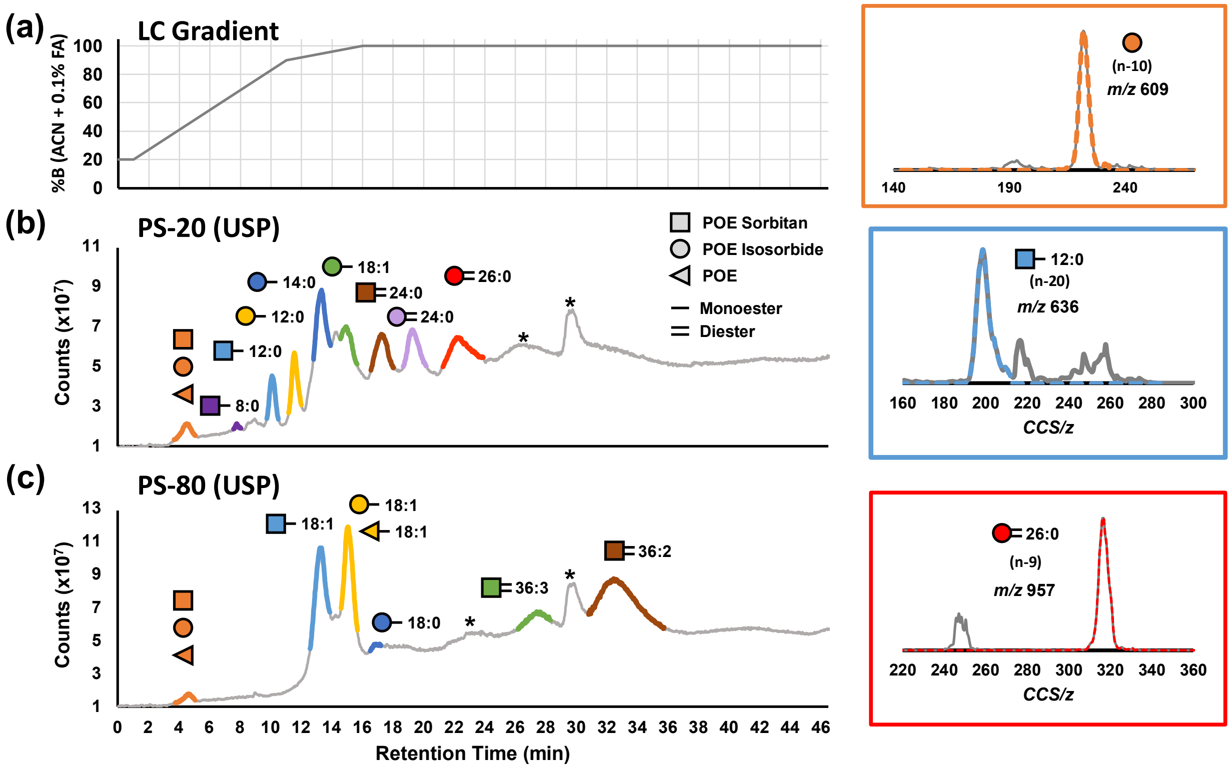

Figure S1. (a) Full 46-minute LC gradient for the developed RPLC method utilizing water with 0.1% formic acid for mobile phase A and acetonitrile with 0.1% formic acid as mobile phase B. Flow rate was set at 400 μL/min and column temperature at 40°C **(b)** Elution of species found in PS-20, with shapes indicating PS series, line(s) representing degree of esterification, and numbers denoting chain length and degree of saturation. Elution order followed the expected trend of more hydrophobic species retaining on column and eluting later. For example, the non-esterified (orange) and smaller monoester (purple) species eluted within the first 8 minutes, followed by species esterified with larger chain lengths (e.g., 12:0-18:1) and diester species (e.g., 24:0 and 28:0). Elution order trends observed in PS-20 were also observed in the PS-80 results **(c)**, though more characteristic PS-80 monoester (e.g., 18:1 and 18:0) and diester (e.g. 36:3 and 36:2) species were found in higher abundance. Panels on the right provided mobility spectrum examples for a given *m/z* window, representing the utility of IM and how it is leveraged to distinguish between overlapping isotopic envelopes and, in some cases, isomeric or isobaric species. The middle panel represents POE Sorbitan monoester 12:0 n-20 adducted with two sodium ions, whereas the others are singly charged. Numerous species were also found within the unannotated TIC peaks (*) but were unable to be confidently identified.

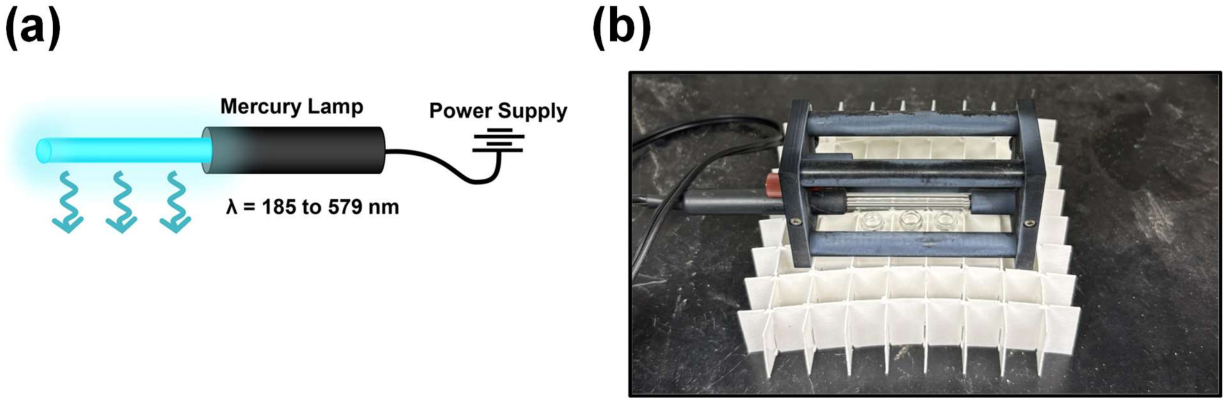

Figure S2. (a) Illustration of mercury lamp utilized for photoirradiation experiments. The specific lamp used has a reported output of 254nm (73 μW/cm^2) and 185 nm (42 μW/cm^2). Figure adapted with permission from Harris et al. 2018. **(b)** Photograph of the custom-built housing for the mercury lamp which stabilizes and secures the lamp into a fixed position and height. Samples are placed below the lamp within a fume hood in the dark to undergo irradiation. During operation, the apparatus is covered to ensure safety and to reduce any external factors (e.g., alternative light sources, contamination) which may affect results.

Table S2. Summary of Mass and Mobility Measurements for PS-20

Table Notes: Values listed in these supplemental tables are *CCS/z* and not CCS. To obtain CCS, multiply the *CCS/z* by the integer charge state. All CCS measurements were obtained in nitrogen drift gas at ambient temperature, which was in the range of 20-28 °C for all experiments.

	Structural Series	Mono-mer	Ion Form	Measurement Replicates	Avg. *m/z*	StDev *m/z*	Theor. *m/z*	Mass Acc. (ppm)	Avg. *CCS/z* (Å²/z)	StDev *CCS/z* (Å²/z)	%RSD *CCS/z* (%)
1	POE Sorbitan	n-17	[M + Na]	5 (x3 Croda, x2 USP)	935.4959	0.0055	935.5040	-2.7	285.80	0.74	0.26%
2	POE Sorbitan	n-18	[M + Na]	5 (x3 Croda, x2 USP)	979.5239	0.0028	979.5300	-0.1	294.17	0.29	0.10%
3	POE Sorbitan	n-19	[M + Na]	5 (x3 Croda, x2 USP)	1023.5506	0.0041	1023.5560	1.1	302.09	0.36	0.12%
4	POE Sorbitan	n-20	[M + Na]	5 (x3 Croda, x2 USP)	1067.5774	0.0038	1067.5830	1.3	309.70	0.21	0.07%
5	POE Sorbitan	n-21	[M + Na]	5 (x3 Croda, x2 USP)	1111.6049	0.0054	1111.6090	3.0	317.34	0.35	0.11%
6	POE Sorbitan	n-22	[M + Na]	5 (x3 Croda, x2 USP)	1155.6293	0.0045	1155.6350	1.9	324.55	0.21	0.06%
7	POE Sorbitan	n-23	[M + Na]	5 (x3 Croda, x2 USP)	1199.6554	0.0047	1199.6610	2.4	331.82	0.25	0.08%
8	POE Sorbitan	n-24	[M + Na]	5 (x3 Croda, x2 USP)	1243.6811	0.0052	1243.6870	2.5	339.26	0.63	0.19%
9	POE Sorbitan	n-25	[M + Na]	5 (x3 Croda, x2 USP)	1287.7054	0.0057	1287.7140	0.6	346.06	0.32	0.09%
10	POE Sorbitan	n-26	[M + Na]	5 (x3 Croda, x2 USP)	1331.7327	0.0057	1331.7400	2.0	353.02	0.22	0.06%
11	POE Sorbitan	n-27	[M + Na]	5 (x3 Croda, x2 USP)	1375.7574	0.0052	1375.7660	1.3	359.25	0.67	0.19%
12	POE Sorbitan	n-28	[M + Na]	5 (x3 Croda, x2 USP)	1419.7849	0.0083	1419.7920	2.6	366.11	1.01	0.28%
13	POE Sorbitan	n-29	[M + Na]	5 (x3 Croda, x2 USP)	1463.8089	0.0069	1463.8180	1.5	373.12	0.58	0.16%
14	POE Sorbitan	n-30	[M + Na]	5 (x3 Croda, x2 USP)	1507.8346	0.0072	1507.8450	0.9	380.23	0.64	0.17%
15	POE Sorbitan	n-15	[M + K]	1 (USP)	863.4197	--	863.4259	-1.7	270.92	--	--
16	POE Sorbitan	n-16	[M + K]	5 (x3 Croda, x2 USP)	907.4449	0.0040	907.4519	-2.0	279.87	0.92	0.33%
17	POE Sorbitan	n-17	[M + K]	5 (x3 Croda, x2 USP)	951.4731	0.0045	951.4779	0.9	287.62	0.33	0.11%
18	POE Sorbitan	n-18	[M + K]	5 (x3 Croda, x2 USP)	995.4977	0.0042	995.5039	0.0	295.49	0.47	0.16%
19	POE Sorbitan	n-19	[M + K]	5 (x3 Croda, x2 USP)	1039.5243	0.0037	1039.5299	1.0	302.91	0.73	0.24%
20	POE Sorbitan	n-20	[M + K]	5 (x3 Croda, x2 USP)	1083.5505	0.0048	1083.5569	0.7	310.52	1.33	0.43%
21	POE Sorbitan	n-21	[M + K]	5 (x3 Croda, x2 USP)	1127.5753	0.0039	1127.5829	0.0	318.35	0.57	0.18%
22	POE Sorbitan	n-22	[M + K]	5 (x3 Croda, x2 USP)	1171.6031	0.0062	1171.6089	2.0	325.70	0.98	0.30%
23	POE Sorbitan	n-23	[M + K]	5 (x3 Croda, x2 USP)	1215.6271	0.0026	1215.6349	0.6	332.97	1.16	0.35%
24	POE Sorbitan	n-24	[M + K]	5 (x3 Croda, x2 USP)	1259.6528	0.0047	1259.6609	0.7	340.34	0.78	0.23%
25	POE Sorbitan	n-25	[M + K]	5 (x3 Croda, x2 USP)	1303.6799	0.0061	1303.6879	1.2	347.24	0.36	0.10%
26	POE Sorbitan	n-26	[M + K]	5 (x3 Croda, x2 USP)	1347.7055	0.0074	1347.7139	1.2	353.96	0.75	0.21%
27	POE Sorbitan	n-27	[M + K]	5 (x3 Croda, x2 USP)	1391.7299	0.0027	1391.7399	0.3	361.13	0.60	0.17%
28	POE Sorbitan monoester 8:0	n-17	[M + Na]	4 (x2 Croda, x2 USP)	1061.5980	0.0069	1061.6083	-3.2	316.88	1.83	0.58%
29	POE Sorbitan monoester 8:0	n-18	[M + Na]	5 (x3 Croda, x2 USP)	1105.6232	0.0049	1105.6345	7.6	324.21	0.35	0.11%
30	POE Sorbitan monoester 8:0	n-19	[M + Na]	5 (x3 Croda, x2 USP)	1149.6514	0.0066	1149.6608	-1.3	331.50	0.60	0.18%
31	POE Sorbitan monoester 8:0	n-20	[M + Na]	5 (x3 Croda, x2 USP)	1193.6767	0.0065	1193.6870	-1.6	338.10	0.53	0.16%
32	POE Sorbitan monoester 8:0	n-21	[M + Na]	5 (x3 Croda, x2 USP)	1237.7031	0.0071	1237.7132	-1.0	345.34	0.60	0.17%
33	POE Sorbitan monoester 8:0	n-22	[M + Na]	5 (x3 Croda, x2 USP)	1281.7271	0.0052	1281.7394	-2.3	351.24	0.64	0.18%
34	POE Sorbitan monoester 8:0	n-23	[M + Na]	5 (x3 Croda, x2 USP)	1325.7518	0.0087	1325.7656	4.7	358.89	0.48	0.13%
35	POE Sorbitan monoester 8:0	n-24	[M + Na]	5 (x3 Croda, x2 USP)	1369.7787	0.0054	1369.7918	-2.0	365.95	0.81	0.22%
36	POE Sorbitan monoester 8:0	n-25	[M + Na]	5 (x3 Croda, x2 USP)	1413.8028	0.0085	1413.8180	3.4	372.93	0.68	0.18%
37	POE Sorbitan monoester 8:0	n-26	[M + Na]	5 (x3 Croda, x2 USP)	1457.8317	0.0117	1457.8443	-0.9	380.59	1.00	0.26%
38	POE Sorbitan monoester 10:0	n-16	[M + Na]	5 (x3 Croda, x2 USP)	1045.5981	0.0085	1045.6134	4.2	315.98	0.71	0.23%
39	POE Sorbitan monoester 10:0	n-17	[M + Na]	5 (x3 Croda, x2 USP)	1089.6243	0.0090	1089.6396	4.1	323.71	1.83	0.56%
40	POE Sorbitan monoester 10:0	n-18	[M + Na]	5 (x3 Croda, x2 USP)	1133.6478	0.0079	1133.6658	1.6	330.57	1.12	0.34%
41	POE Sorbitan monoester 10:0	n-19	[M + Na]	5 (x3 Croda, x2 USP)	1177.6736	0.0068	1177.6921	1.2	337.98	1.23	0.36%
42	POE Sorbitan monoester 10:0	n-20	[M + Na]	5 (x3 Croda, x2 USP)	1221.6993	0.0079	1221.7183	0.7	344.43	0.45	0.13%
43	POE Sorbitan monoester 10:0	n-21	[M + Na]	5 (x3 Croda, x2 USP)	1265.7256	0.0088	1265.7445	0.8	351.62	0.62	0.18%
44	POE Sorbitan monoester 10:0	n-22	[M + Na]	5 (x3 Croda, x2 USP)	1309.7493	0.0079	1309.7707	-1.1	358.35	0.17	0.05%
45	POE Sorbitan monoester 10:0	n-23	[M + Na]	5 (x3 Croda, x2 USP)	1353.7751	0.0081	1353.7969	-1.3	365.08	0.52	0.14%
46	POE Sorbitan monoester 10:0	n-24	[M + Na]	5 (x3 Croda, x2 USP)	1397.8002	0.0078	1397.8231	-2.1	371.40	0.31	0.08%
47	POE Sorbitan monoester 10:0	n-25	[M + Na]	5 (x3 Croda, x2 USP)	1441.8242	0.0073	1441.8493	-3.5	378.54	0.82	0.22%
48	POE Sorbitan monoester 10:0	n-26	[M + Na]	5 (x3 Croda, x2 USP)	1485.8509	0.0087	1485.8756	-3.0	384.23	0.81	0.21%
49	POE Sorbitan monoester 10:0	n-27	[M + Na]	5 (x3 Croda, x2 USP)	1529.8759	0.0072	1529.9018	-3.7	390.58	1.12	0.29%
50	POE Sorbitan monoester 10:0	n-28	[M + Na]	5 (x3 Croda, x2 USP)	1573.9005	0.0096	1573.9280	-4.6	397.73	0.94	0.24%
51	POE Sorbitan monoester 10:0	n-29	[M + Na]	5 (x3 Croda, x2 USP)	1617.9278	0.0103	1617.9542	-3.8	403.86	0.86	0.21%
52	POE Sorbitan monoester 10:0	n-30	[M + Na]	5 (x3 Croda, x2 USP)	1661.9534	0.0094	1661.9804	-4.1	410.72	1.21	0.29%
53	POE Sorbitan monoester 12:0	n-15	[M + Na]	5 (x3 Croda, x2 USP)	1029.6144	0.0509	1029.6185	2.4	314.08	0.40	0.13%
54	POE Sorbitan monoester 12:0	n-16	[M + Na]	5 (x3 Croda, x2 USP)	1073.6303	0.0068	1073.6447	4.9	321.92	0.24	0.08%
55	POE Sorbitan monoester 12:0	n-17	[M + Na]	5 (x3 Croda, x2 USP)	1117.6609	0.0054	1117.6709	-2.2	328.94	0.42	0.13%
56	POE Sorbitan monoester 12:0	n-18	[M + Na]	5 (x3 Croda, x2 USP)	1161.6890	0.0046	1161.6971	-0.1	336.24	0.26	0.08%
57	POE Sorbitan monoester 12:0	n-19	[M + Na]	5 (x3 Croda, x2 USP)	1205.7153	0.0049	1205.7234	0.3	343.16	0.38	0.11%
58	POE Sorbitan monoester 12:0	n-20	[M + Na]	5 (x3 Croda, x2 USP)	1249.7422	0.0054	1249.7496	1.3	350.22	0.23	0.06%
59	POE Sorbitan monoester 12:0	n-21	[M + Na]	5 (x3 Croda, x2 USP)	1293.7687	0.0049	1293.7758	1.8	357.12	0.22	0.06%
60	POE Sorbitan monoester 12:0	n-22	[M + Na]	5 (x3 Croda, x2 USP)	1337.7939	0.0057	1337.8020	1.4	363.98	0.23	0.06%
61	POE Sorbitan monoester 12:0	n-23	[M + Na]	5 (x3 Croda, x2 USP)	1381.8194	0.0066	1381.8282	1.2	370.55	0.20	0.05%
62	POE Sorbitan monoester 12:0	n-24	[M + Na]	5 (x3 Croda, x2 USP)	1425.8447	0.0069	1425.8544	0.8	377.20	0.36	0.09%
63	POE Sorbitan monoester 12:0	n-25	[M + Na]	5 (x3 Croda, x2 USP)	1469.8705	0.0060	1469.8806	0.9	383.08	0.32	0.08%
64	POE Sorbitan monoester 12:0	n-26	[M + Na]	5 (x3 Croda, x2 USP)	1513.8955	0.0075	1513.9068	0.3	390.12	0.34	0.09%
65	POE Sorbitan monoester 12:0	n-27	[M + Na]	5 (x3 Croda, x2 USP)	1557.9213	0.0066	1557.9331	0.4	396.36	0.48	0.12%
66	POE Sorbitan monoester 12:0	n-28	[M + Na]	5 (x3 Croda, x2 USP)	1601.9471	0.0066	1601.9593	0.4	402.83	0.71	0.18%
67	POE Sorbitan monoester 12:0	n-29	[M + Na]	5 (x3 Croda, x2 USP)	1645.9725	0.0065	1645.9855	0.2	409.41	0.99	0.24%

= Signal Interference from Neighboring Isotopic Envelope(s)
= Low Signal Abundance and/or Poor Feature Resolution

Table S2. (continued)

	Structural Series	Mono-mer	Ion Form	Measurement Replicates	Avg. *m/z*	StDev *m/z*	Theor. *m/z*	Mass Acc. (ppm)	Avg. *CCS/z* (Å²/z)	StDev *CCS/z* (Å²/z)	%RSD *CCS/z* (%)
68	POE Sorbitan monoester 12:0	n-30	[M + Na]	5 (x3 Croda, x2 USP)	1689.9969	0.0099	1690.0117	-0.6	414.55	1.00	0.24%
69	POE Sorbitan monoester 12:0	n-31	[M + Na]	5 (x3 Croda, x2 USP)	1734.0249	0.0077	1734.0379	0.7	421.28	1.49	0.35%
70	POE Sorbitan monoester 14:0	n-19	[M + Na]	5 (x3 Croda, x2 USP)	1233.7461	0.0051	1233.7547	0.2	348.85	0.43	0.12%
71	POE Sorbitan monoester 14:0	n-20	[M + Na]	5 (x3 Croda, x2 USP)	1277.7726	0.0050	1277.7809	0.8	355.97	0.32	0.09%
72	POE Sorbitan monoester 14:0	n-21	[M + Na]	5 (x3 Croda, x2 USP)	1321.7981	0.0064	1321.8071	0.6	362.53	0.25	0.07%
73	POE Sorbitan monoester 14:0	n-22	[M + Na]	5 (x3 Croda, x2 USP)	1365.8231	0.0070	1365.8333	0.1	369.26	0.33	0.09%
74	POE Sorbitan monoester 14:0	n-23	[M + Na]	5 (x3 Croda, x2 USP)	1409.8479	0.0068	1409.8595	-0.6	375.41	0.75	0.20%
75	POE Sorbitan monoester 14:0	n-24	[M + Na]	5 (x3 Croda, x2 USP)	1453.8737	0.0079	1453.8857	-0.6	382.11	0.41	0.11%
76	POE Sorbitan monoester 14:0	n-25	[M + Na]	5 (x3 Croda, x2 USP)	1497.9002	0.0084	1497.9119	0.0	389.04	0.67	0.17%
77	POE Sorbitan monoester 14:0	n-26	[M + Na]	5 (x3 Croda, x2 USP)	1541.9238	0.0079	1541.9381	-1.4	395.68	0.62	0.16%
78	POE Sorbitan monoester 14:0	n-27	[M + Na]	5 (x3 Croda, x2 USP)	1585.9517	0.0060	1585.9644	0.0	402.06	0.78	0.19%
79	POE Sorbitan monoester 14:0	n-28	[M + Na]	5 (x3 Croda, x2 USP)	1629.9762	0.0060	1629.9906	-0.8	408.37	1.53	0.37%
80	POE Sorbitan monoester 14:0	n-29	[M + Na]	5 (x3 Croda, x2 USP)	1673.9995	0.0116	1674.0168	1.8	413.96	0.81	0.20%
81	POE Sorbitan monoester 16:0	n-25	[M + Na]	4 (x2 Croda, x2 USP)	1525.9281	0.0057	1525.9432	-2.1	395.04	0.73	0.18%
82	POE Sorbitan monoester 16:0	n-26	[M + Na]	5 (x3 Croda, x2 USP)	1569.9513	0.0118	1569.9694	1.3	399.24	1.31	0.33%
83	POE Sorbitan monoester 16:0	n-27	[M + Na]	5 (x3 Croda, x2 USP)	1613.9766	0.0111	1613.9957	0.7	406.53	0.87	0.21%
84	POE Sorbitan monoester 18:1	n-7	[M + Na]	5 (x3 Croda, x2 USP)	759.4830	0.0032	759.4870	-0.4	273.13	0.72	0.26%
85	POE Sorbitan monoester 18:1	n-8	[M + Na]	5 (x3 Croda, x2 USP)	803.5096	0.0044	803.5132	0.6	281.02	0.62	0.22%
86	POE Sorbitan monoester 18:1	n-9	[M + Na]	5 (x3 Croda, x2 USP)	847.5356	0.0042	847.5394	0.9	288.22	0.49	0.17%
87	POE Sorbitan monoester 18:1	n-10	[M + Na]	5 (x3 Croda, x2 USP)	891.5612	0.0039	891.5657	0.7	295.66	0.40	0.14%
88	POE Sorbitan monoester 18:1	n-11	[M + Na]	5 (x3 Croda, x2 USP)	935.5864	0.0035	935.5919	0.1	302.96	0.20	0.06%
89	POE Sorbitan monoester 18:1	n-12	[M + Na]	5 (x3 Croda, x2 USP)	979.6120	0.0050	979.6181	0.0	310.56	0.76	0.25%
90	POE Sorbitan monoester 18:1	n-13	[M + Na]	5 (x3 Croda, x2 USP)	1023.6358	0.0041	1023.6443	-2.0	317.41	0.63	0.20%
91	POE Sorbitan monoester 18:1	n-14	[M + Na]	5 (x3 Croda, x2 USP)	1067.6613	0.0054	1067.6705	-2.1	325.18	1.03	0.32%
92	POE Sorbitan monoester 18:1	n-15	[M + Na]	5 (x3 Croda, x2 USP)	1111.6851	0.0028	1111.6967	7.4	331.85	0.62	0.19%
93	POE Sorbitan monoester 18:1	n-16	[M + Na]	5 (x3 Croda, x2 USP)	1155.7120	0.0049	1155.7230	-2.6	339.56	0.81	0.24%
94	POE Sorbitan monoester 18:1	n-17	[M + Na]	5 (x3 Croda, x2 USP)	1199.7402	0.0053	1199.7492	-0.4	346.69	1.31	0.38%
95	POE Sorbitan monoester 18:1	n-18	[M + Na]	5 (x3 Croda, x2 USP)	1243.7637	0.0017	1243.7754	-2.3	352.49	1.15	0.33%
96	POE Sorbitan monoester 18:1	n-19	[M + Na]	5 (x3 Croda, x2 USP)	1287.7893	0.0048	1287.8016	-2.3	360.35	0.21	0.06%
97	POE Sorbitan monoester 18:1	n-20	[M + Na]	5 (x3 Croda, x2 USP)	1331.8164	0.0039	1331.8278	-1.2	367.46	0.60	0.16%
98	POE Sorbitan monoester 18:1	n-21	[M + Na]	5 (x3 Croda, x2 USP)	1375.8446	0.0087	1375.8540	0.7	374.40	0.79	0.21%
99	POE Sorbitan monoester 18:1	n-22	[M + Na]	5 (x3 Croda, x2 USP)	1419.8674	0.0068	1419.8802	-1.4	380.89	1.05	0.28%
100	POE Sorbitan monoester 18:1	n-23	[M + Na]	5 (x3 Croda, x2 USP)	1463.8917	0.0063	1463.9065	3.6	387.92	0.47	0.12%
101	POE Sorbitan monoester 18:1	n-24	[M + Na]	5 (x3 Croda, x2 USP)	1507.9158	0.0054	1507.9327	2.2	395.14	0.89	0.23%
102	POE Sorbitan monoester 18:1	n-25	[M + Na]	5 (x3 Croda, x2 USP)	1551.9448	0.0063	1551.9589	-1.2	402.09	1.21	0.30%
103	POE Sorbitan monoester 18:1	n-26	[M + Na]	4 (x2 Croda, x2 USP)	1595.9711	0.0075	1595.9851	-0.8	406.97	0.64	0.16%
104	POE Sorbitan monoester 18:1	n-27	[M + Na]	5 (x3 Croda, x2 USP)	1639.9948	0.0101	1640.0113	2.3	413.16	1.94	0.47%
105	POE Sorbitan monoester 18:1	n-28	[M + Na]	5 (x3 Croda, x2 USP)	1684.0199	0.0101	1684.0375	1.6	421.12	0.76	0.18%
106	POE Sorbitan monoester 18:1	n-29	[M + Na]	5 (x3 Croda, x2 USP)	1728.0431	0.0112	1728.0637	-0.2	426.02	1.32	0.31%
107	POE Sorbitan diester 22:0	n-15	[M + Na]	5 (x3 Croda, x2 USP)	1183.7453	0.0088	1183.7543	-0.6	343.50	0.67	0.20%
108	POE Sorbitan diester 22:0	n-16	[M + Na]	5 (x3 Croda, x2 USP)	1227.7720	0.0110	1227.7805	0.2	351.73	0.83	0.24%
109	POE Sorbitan diester 22:0	n-17	[M + Na]	5 (x3 Croda, x2 USP)	1271.7945	0.0062	1271.8067	-2.4	359.85	1.43	0.40%
110	POE Sorbitan diester 22:0	n-18	[M + Na]	5 (x3 Croda, x2 USP)	1315.8149	0.0072	1315.8329	1.5	366.67	1.45	0.40%
111	POE Sorbitan diester 22:0	n-19	[M + Na]	5 (x3 Croda, x2 USP)	1359.8427	0.0092	1359.8591	2.7	374.87	0.74	0.20%
112	POE Sorbitan diester 22:0	n-20	[M + Na]	5 (x3 Croda, x2 USP)	1403.8692	0.0103	1403.8853	2.8	381.06	0.97	0.25%
113	POE Sorbitan diester 22:0	n-21	[M + Na]	5 (x3 Croda, x2 USP)	1447.8919	0.0080	1447.9115	0.3	388.16	0.91	0.24%
114	POE Sorbitan diester 22:0	n-22	[M + Na]	5 (x3 Croda, x2 USP)	1491.9166	0.0076	1491.9377	-0.7	394.45	0.77	0.20%
115	POE Sorbitan diester 22:0	n-23	[M + Na]	5 (x3 Croda, x2 USP)	1535.9422	0.0089	1535.9640	-1.1	400.83	0.67	0.17%
116	POE Sorbitan diester 22:0	n-24	[M + Na]	5 (x3 Croda, x2 USP)	1579.9691	0.0095	1579.9902	-0.6	407.18	2.22	0.54%
117	POE Sorbitan diester 22:0	n-25	[M + Na]	5 (x3 Croda, x2 USP)	1623.9934	0.0093	1624.0164	-1.7	413.53	1.53	0.37%
118	POE Sorbitan diester 22:0	n-26	[M + Na]	5 (x3 Croda, x2 USP)	1668.0244	0.0138	1668.0426	1.2	419.52	1.23	0.29%
119	POE Sorbitan diester 22:0	n-27	[M + Na]	5 (x3 Croda, x2 USP)	1712.0449	0.0117	1712.0688	-2.1	425.55	0.76	0.18%
120	POE Sorbitan diester 22:0	n-28	[M + Na]	5 (x3 Croda, x2 USP)	1756.0735	0.0134	1756.0950	-0.6	431.83	1.51	0.35%
121	POE Sorbitan diester 24:0	n-17	[M + Na]	5 (x3 Croda, x2 USP)	1299.8242	0.0056	1299.8380	4.7	366.34	1.45	0.40%
122	POE Sorbitan diester 24:0	n-18	[M + Na]	5 (x3 Croda, x2 USP)	1343.8518	0.0065	1343.8642	-1.8	373.76	0.38	0.10%
123	POE Sorbitan diester 24:0	n-19	[M + Na]	5 (x3 Croda, x2 USP)	1387.8790	0.0067	1387.8904	-0.7	380.12	0.61	0.16%
124	POE Sorbitan diester 24:0	n-20	[M + Na]	5 (x3 Croda, x2 USP)	1431.9048	0.0060	1431.9166	-0.6	386.62	0.43	0.11%
125	POE Sorbitan diester 24:0	n-21	[M + Na]	5 (x3 Croda, x2 USP)	1475.9303	0.0072	1475.9428	-0.7	393.62	0.66	0.17%
126	POE Sorbitan diester 24:0	n-22	[M + Na]	5 (x3 Croda, x2 USP)	1519.9556	0.0085	1519.9691	-1.0	399.38	0.74	0.19%
127	POE Sorbitan diester 24:0	n-23	[M + Na]	5 (x3 Croda, x2 USP)	1563.9807	0.0083	1563.9953	-1.4	405.50	0.58	0.14%
128	POE Sorbitan diester 24:0	n-24	[M + Na]	5 (x3 Croda, x2 USP)	1608.0077	0.0060	1608.0215	-0.5	412.00	0.73	0.18%
129	POE Sorbitan diester 24:0	n-25	[M + Na]	5 (x3 Croda, x2 USP)	1652.0317	0.0081	1652.0477	-1.6	418.29	0.76	0.18%
130	POE Sorbitan diester 24:0	n-26	[M + Na]	5 (x3 Croda, x2 USP)	1696.0562	0.0071	1696.0739	1.5	424.60	1.31	0.31%
131	POE Sorbitan diester 24:0	n-27	[M + Na]	5 (x3 Croda, x2 USP)	1740.0831	0.0094	1740.1001	-1.6	430.15	0.86	0.20%
132	POE Sorbitan diester 24:0	n-28	[M + Na]	4 (x3 Croda, x1 USP)	1784.1015	0.0114	1784.1263	-2.5	434.74	0.63	0.14%
133	POE Sorbitan diester 24:0	n-29	[M + Na]	4 (x3 Croda, x1 USP)	1828.1234	0.0092	1828.1525	-4.8	441.63	1.66	0.38%
134	POE Sorbitan diester 24:0	n-30	[M + Na]	4 (x3 Croda, x1 USP)	1872.1523	0.0106	1872.1788	-3.2	446.84	1.36	0.30%

= Signal Interference from Neighboring Isotopic Envelope(s)
= Low Signal Abundance and/or Poor Feature Resolution

Table S2. (continued)

	Structural Series	Mono-mer	Ion Form	Measurement Replicates	Avg. *m/z*	StDev *m/z*	Theor. *m/z*	Mass Acc. (ppm)	Avg. *CCS/z* (Å^2/z)	StDev *CCS/z* (Å^2/z)	%RSD *CCS/z* (%)
135	POE Sorbitan diester 26:0	n-23	[M + Na]	5 (x3 Croda, x2 USP)	1592.0148	0.0107	1592.0266	0.6	410.44	0.56	0.14%
136	POE Sorbitan diester 26:0	n-24	[M + Na]	5 (x3 Croda, x2 USP)	1636.0401	0.0060	1636.0528	0.3	417.53	1.56	0.37%
137	POE Sorbitan diester 26:0	n-25	[M + Na]	5 (x3 Croda, x2 USP)	1680.0625	0.0081	1680.0790	-1.7	423.31	1.89	0.45%
138	POE Sorbitan diester 26:0	n-26	[M + Na]	5 (x3 Croda, x2 USP)	1724.0864	0.0083	1724.1052	0.9	428.80	1.23	0.29%
139	POE Sorbitan diester 26:0	n-27	[M + Na]	5 (x3 Croda, x2 USP)	1768.1121	0.0109	1768.1314	0.6	434.48	0.68	0.16%
140	POE Sorbitan diester 26:0	n-28	[M + Na]	4 (x3 Croda, x1 USP)	1812.1305	0.0076	1812.1576	-3.7	439.44	1.65	0.38%
141	POE Sorbitan diester 28:0	n-24	[M + Na]	5 (x3 Croda, x2 USP)	1664.0707	0.0115	1664.0841	0.1	421.21	0.95	0.23%
142	POE Sorbitan diester 28:0	n-25	[M + Na]	5 (x3 Croda, x2 USP)	1708.0941	0.0096	1708.1103	-1.3	427.14	1.16	0.27%
143	POE Sorbitan diester 28:0	n-26	[M + Na]	5 (x3 Croda, x2 USP)	1752.1206	0.0108	1752.1365	-0.8	434.50	0.77	0.18%
144	POE Sorbitan diester 28:0	n-27	[M + Na]	4 (x3 Croda, x1 USP)	1796.1372	0.0100	1796.1627	-2.9	440.53	2.47	0.56%
145	POE Sorbitan diester 28:0	n-28	[M + Na]	4 (x3 Croda, x1 USP)	1840.1675	0.0132	1840.1889	-0.5	445.42	1.79	0.40%
146	POE Sorbitan monoester 8:0	n-13	[M + 2Na]	5 (x3 Croda, x2 USP)	454.2201	0.0034	454.2466	-16.1	151.64	0.49	0.32%
147	POE Sorbitan monoester 8:0	n-14	[M + 2Na]	5 (x3 Croda, x2 USP)	476.2412	0.0112	476.2597	1.5	156.13	1.00	0.64%
148	POE Sorbitan monoester 8:0	n-15	[M + 2Na]	5 (x3 Croda, x2 USP)	498.2515	0.0066	498.2728	-4.2	159.10	0.53	0.33%
149	POE Sorbitan monoester 8:0	n-16	[M + 2Na]	5 (x3 Croda, x2 USP)	520.2634	0.0029	520.2859	-6.2	162.13	0.99	0.61%
150	POE Sorbitan monoester 8:0	n-17	[M + 2Na]	5 (x3 Croda, x2 USP)	542.2753	0.0017	542.2991	-8.3	166.03	0.44	0.27%
151	POE Sorbitan monoester 8:0	n-18	[M + 2Na]	5 (x3 Croda, x2 USP)	564.2880	0.0021	564.3122	-8.6	169.34	0.31	0.19%
152	POE Sorbitan monoester 8:0	n-19	[M + 2Na]	5 (x3 Croda, x2 USP)	586.3016	0.0023	586.3253	-7.3	172.63	0.47	0.27%
153	POE Sorbitan monoester 8:0	n-20	[M + 2Na]	5 (x3 Croda, x2 USP)	608.3176	0.0085	608.3384	-2.4	176.00	0.32	0.18%
154	POE Sorbitan monoester 8:0	n-21	[M + 2Na]	5 (x3 Croda, x2 USP)	630.3280	0.0020	630.3515	-6.5	179.42	0.20	0.11%
155	POE Sorbitan monoester 8:0	n-22	[M + 2Na]	5 (x3 Croda, x2 USP)	652.3403	0.0023	652.3646	-7.5	182.58	0.56	0.31%
156	POE Sorbitan monoester 8:0	n-23	[M + 2Na]	5 (x3 Croda, x2 USP)	674.3540	0.0022	674.3777	-6.3	185.82	0.61	0.33%
157	POE Sorbitan monoester 8:0	n-24	[M + 2Na]	5 (x3 Croda, x2 USP)	696.3652	0.0037	696.3908	-8.8	188.73	0.41	0.22%
158	POE Sorbitan monoester 8:0	n-25	[M + 2Na]	5 (x3 Croda, x2 USP)	718.3781	0.0026	718.4039	-8.9	192.03	0.63	0.33%
159	POE Sorbitan monoester 8:0	n-26	[M + 2Na]	5 (x3 Croda, x2 USP)	740.3931	0.0042	740.4170	-6.0	195.27	0.67	0.34%
160	POE Sorbitan monoester 8:0	n-27	[M + 2Na]	5 (x3 Croda, x2 USP)	762.4045	0.0055	762.4301	-8.1	198.31	0.40	0.20%
161	POE Sorbitan monoester 8:0	n-28	[M + 2Na]	4 (x2 Croda, x2 USP)	784.4205	0.0012	784.4432	-4.1	201.58	0.49	0.24%
162	POE Sorbitan monoester 8:0	n-29	[M + 2Na]	2 (USP)	806.4344	0.0031	806.4563	-3.0	205.57	0.35	0.17%
163	POE Sorbitan monoester 12:0	n-19	[M + 2Na]	5 (x3 Croda, x2 USP)	614.3541	0.0030	614.3566	-0.6	193.10	1.03	0.53%
164	POE Sorbitan monoester 12:0	n-20	[M + 2Na]	5 (x3 Croda, x2 USP)	636.3677	0.0030	636.3697	0.5	197.37	0.85	0.43%
165	POE Sorbitan monoester 12:0	n-21	[M + 2Na]	5 (x3 Croda, x2 USP)	658.3800	0.0026	658.3828	-0.4	200.81	0.54	0.27%
166	POE Sorbitan monoester 12:0	n-22	[M + 2Na]	5 (x3 Croda, x2 USP)	680.3931	0.0034	680.3959	0.0	204.63	0.55	0.27%
167	POE Sorbitan monoester 12:0	n-23	[M + 2Na]	5 (x3 Croda, x2 USP)	702.4052	0.0027	702.4090	-1.0	208.06	0.74	0.35%
168	POE Sorbitan monoester 12:0	n-24	[M + 2Na]	5 (x3 Croda, x2 USP)	724.4190	0.0028	724.4221	0.2	211.28	0.80	0.38%
169	POE Sorbitan monoester 12:0	n-25	[M + 2Na]	5 (x3 Croda, x2 USP)	746.4322	0.0032	746.4352	0.7	214.51	0.90	0.42%
170	POE Sorbitan monoester 12:0	n-26	[M + 2Na]	5 (x3 Croda, x2 USP)	768.4454	0.0033	768.4483	1.1	217.92	0.78	0.36%
171	POE Sorbitan monoester 12:0	n-27	[M + 2Na]	5 (x3 Croda, x2 USP)	790.4588	0.0048	790.4614	1.7	221.10	0.81	0.36%
172	POE Sorbitan monoester 12:0	n-28	[M + 2Na]	5 (x3 Croda, x2 USP)	812.4708	0.0032	812.4745	0.7	224.13	0.74	0.33%
173	POE Sorbitan monoester 12:0	n-29	[M + 2Na]	5 (x3 Croda, x2 USP)	834.4852	0.0037	834.4876	2.4	227.03	0.82	0.36%
174	POE Sorbitan monoester 12:0	n-30	[M + 2Na]	5 (x3 Croda, x2 USP)	856.4998	0.0043	856.5007	4.3	230.45	0.62	0.27%
175	POE Sorbitan monoester 12:0	n-31	[M + 2Na]	5 (x3 Croda, x2 USP)	878.5150	0.0043	878.5138	6.9	233.51	0.70	0.30%
176	POE Sorbitan monoester 12:0	n-32	[M + 2Na]	5 (x3 Croda, x2 USP)	900.5301	0.0071	900.5269	9.3	236.33	1.08	0.46%
177	POE Sorbitan diester 20:0	n-15	[M + 2Na]	4 (x2 Croda, x2 USP)	589.3317	0.0028	589.3564	-9.2	179.66	0.57	0.32%
178	POE Sorbitan diester 20:0	n-16	[M + 2Na]	5 (x3 Croda, x2 USP)	611.3480	0.0055	611.3695	-3.5	182.35	0.69	0.38%
179	POE Sorbitan diester 20:0	n-17	[M + 2Na]	5 (x3 Croda, x2 USP)	633.3599	0.0011	633.3826	-5.3	185.49	0.47	0.26%
180	POE Sorbitan diester 20:0	n-18	[M + 2Na]	5 (x3 Croda, x2 USP)	655.3733	0.0037	655.3957	-4.6	188.48	0.57	0.30%
181	POE Sorbitan diester 20:0	n-19	[M + 2Na]	5 (x3 Croda, x2 USP)	677.3855	0.0029	677.4088	-5.7	191.63	0.62	0.32%
182	POE Sorbitan diester 20:0	n-20	[M + 2Na]	5 (x3 Croda, x2 USP)	699.3976	0.0018	699.4219	-7.0	194.81	0.49	0.25%
183	POE Sorbitan diester 20:0	n-21	[M + 2Na]	5 (x3 Croda, x2 USP)	721.4126	0.0035	721.4350	-4.1	197.82	0.57	0.29%
184	POE Sorbitan diester 20:0	n-22	[M + 2Na]	5 (x3 Croda, x2 USP)	743.4262	0.0043	743.4481	-3.3	200.88	0.54	0.27%
185	POE Sorbitan diester 20:0	n-23	[M + 2Na]	5 (x3 Croda, x2 USP)	765.4404	0.0078	765.4612	-1.8	203.91	0.66	0.32%
186	POE Sorbitan diester 20:0	n-24	[M + 2Na]	5 (x3 Croda, x2 USP)	787.4497	0.0034	787.4743	-6.5	206.75	0.87	0.42%
187	POE Sorbitan diester 20:0	n-25	[M + 2Na]	5 (x3 Croda, x2 USP)	809.4623	0.0027	809.4874	-6.9	209.77	0.58	0.27%
188	POE Sorbitan diester 20:0	n-26	[M + 2Na]	5 (x3 Croda, x2 USP)	831.4765	0.0013	831.5005	-5.5	213.13	0.86	0.40%
189	POE Sorbitan diester 20:0	n-27	[M + 2Na]	4 (x2 Croda, x2 USP)	853.4873	0.0053	853.5136	-8.0	215.42	1.00	0.46%
190	POE Sorbitan diester 20:0	n-28	[M + 2Na]	4 (x2 Croda, x2 USP)	875.5037	0.0068	875.5268	-3.9	218.36	0.90	0.41%
191	POE Sorbitan diester 28:0	n-25	[M + 2Na]	5 (x3 Croda, x2 USP)	865.5421	0.0060	865.5500	-3.6	233.28	1.05	0.45%
192	POE Sorbitan diester 28:0	n-26	[M + 2Na]	5 (x3 Croda, x2 USP)	887.5587	0.0105	887.5631	0.7	236.47	0.82	0.35%
193	POE Sorbitan diester 28:0	n-27	[M + 2Na]	5 (x3 Croda, x2 USP)	909.5689	0.0048	909.5762	-2.3	239.11	1.00	0.42%
194	POE Sorbitan diester 28:0	n-28	[M + 2Na]	5 (x3 Croda, x2 USP)	931.5847	0.0072	931.5894	1.0	242.53	0.86	0.35%
195	POE Sorbitan diester 28:0	n-29	[M + 2Na]	5 (x3 Croda, x2 USP)	953.5961	0.0068	953.6025	-0.7	245.39	0.89	0.36%
196	POE Sorbitan diester 28:0	n-30	[M + 2Na]	5 (x3 Croda, x2 USP)	975.6127	0.0087	975.6156	3.2	248.34	0.77	0.31%
197	POE Sorbitan diester 28:0	n-31	[M + 2Na]	5 (x3 Croda, x2 USP)	997.6221	0.0062	997.6287	-0.3	251.48	0.67	0.27%
198	POE Sorbitan diester 28:0	n-32	[M + 2Na]	5 (x3 Croda, x2 USP)	1019.6391	0.0105	1019.6418	3.7	254.49	0.62	0.25%

= Signal Interference from Neighboring Isotopic Envelope(s)
= Low Signal Abundance and/or Poor Feature Resolution

Table S2. (continued)

	Structural Series	Monomer	Ion Form	Measurement Replicates	Avg. *m/z*	StDev *m/z*	Theor. *m/z*	Mass Acc. (ppm)	Avg. *CCS/z* (Å²/z)	StDev *CCS/z* (Å²/z)	%RSD *CCS/z* (%)
199	POE Isosorbide	n-6	[M + Na]	5 (x3 Croda, x2 USP)	433.2025	0.0016	433.2050	-5.5	186.70	0.40	0.21%
200	POE Isosorbide	n-7	[M + Na]	5 (x3 Croda, x2 USP)	477.2299	0.0018	477.2312	-1.5	195.14	0.29	0.15%
201	POE Isosorbide	n-8	[M + Na]	5 (x3 Croda, x2 USP)	521.2559	0.0021	521.2574	-0.8	204.37	0.26	0.13%
202	POE Isosorbide	n-9	[M + Na]	5 (x3 Croda, x2 USP)	565.2824	0.0022	565.2836	0.5	213.27	0.22	0.11%
203	POE Isosorbide	n-10	[M + Na]	5 (x3 Croda, x2 USP)	609.3080	0.0024	609.3098	0.3	221.91	0.28	0.13%
204	POE Isosorbide	n-11	[M + Na]	5 (x3 Croda, x2 USP)	653.3344	0.0024	653.3360	1.4	231.10	0.23	0.10%
205	POE Isosorbide	n-12	[M + Na]	5 (x3 Croda, x2 USP)	697.3604	0.0028	697.3622	1.7	240.52	0.24	0.10%
206	POE Isosorbide	n-13	[M + Na]	5 (x3 Croda, x2 USP)	741.3864	0.0032	741.3885	1.8	249.84	0.22	0.09%
207	POE Isosorbide	n-14	[M + Na]	5 (x3 Croda, x2 USP)	785.4126	0.0030	785.4147	2.4	258.92	0.21	0.08%
208	POE Isosorbide	n-15	[M + Na]	5 (x3 Croda, x2 USP)	829.4388	0.0036	829.4409	2.8	267.71	0.16	0.06%
209	POE Isosorbide	n-16	[M + Na]	5 (x3 Croda, x2 USP)	873.4644	0.0029	873.4671	2.6	276.25	0.20	0.07%
210	POE Isosorbide	n-17	[M + Na]	5 (x3 Croda, x2 USP)	917.4892	0.0036	917.4933	1.3	284.51	0.25	0.09%
211	POE Isosorbide	n-18	[M + Na]	5 (x3 Croda, x2 USP)	961.5144	0.0035	961.5195	0.8	292.55	0.32	0.11%
212	POE Isosorbide	n-19	[M + Na]	5 (x3 Croda, x2 USP)	1005.5399	0.0041	1005.5460	0.2	300.50	0.31	0.10%
213	POE Isosorbide	n-8	[M + K]	5 (x3 Croda, x2 USP)	537.2282	0.0021	537.2319	-4.7	207.14	0.54	0.26%
214	POE Isosorbide	n-9	[M + K]	5 (x3 Croda, x2 USP)	581.2556	0.0017	581.2579	-1.2	215.45	0.28	0.13%
215	POE Isosorbide	n-10	[M + K]	5 (x3 Croda, x2 USP)	625.2813	0.0022	625.2839	-0.7	223.84	0.20	0.09%
216	POE Isosorbide	n-11	[M + K]	5 (x3 Croda, x2 USP)	669.3079	0.0022	669.3099	0.9	232.47	0.18	0.08%
217	POE Isosorbide	n-12	[M + K]	5 (x3 Croda, x2 USP)	713.3339	0.0025	713.3359	1.5	241.51	0.22	0.09%
218	POE Isosorbide	n-13	[M + K]	5 (x3 Croda, x2 USP)	757.3599	0.0024	757.3619	2.0	250.76	0.17	0.07%
219	POE Isosorbide	n-14	[M + K]	5 (x3 Croda, x2 USP)	801.3861	0.0020	801.3879	2.8	259.76	0.12	0.05%
220	POE Isosorbide	n-15	[M + K]	5 (x3 Croda, x2 USP)	845.4117	0.0030	845.4139	2.7	268.66	0.13	0.05%
221	POE Isosorbide	n-16	[M + K]	5 (x3 Croda, x2 USP)	889.4370	0.0027	889.4399	2.3	277.66	0.28	0.10%
222	POE Isosorbide	n-17	[M + K]	5 (x3 Croda, x2 USP)	933.4625	0.0036	933.4659	2.2	285.85	0.43	0.15%
223	POE Isosorbide	n-18	[M + K]	5 (x3 Croda, x2 USP)	977.4881	0.0042	977.4919	2.2	293.82	0.74	0.25%
224	POE Isosorbide	n-19	[M + K]	5 (x3 Croda, x2 USP)	1021.5132	0.0029	1021.5179	1.7	302.15	0.44	0.15%
225	POE Isosorbide	n-20	[M + K]	5 (x3 Croda, x2 USP)	1065.5409	0.0048	1065.5439	3.7	310.36	0.90	0.29%
226	POE Isosorbide	n-21	[M + K]	4 (x2 Croda, x2 USP)	1109.5848	0.0382	1109.5699	20.1	317.41	1.79	0.56%
227	POE Isosorbide monoester 10:0	n-8	[M + Na]	5 (x3 Croda, x2 USP)	675.3858	0.0051	675.3931	17.8	251.19	0.99	0.39%
228	POE Isosorbide monoester 10:0	n-9	[M + Na]	5 (x3 Croda, x2 USP)	719.4083	0.0050	719.4194	11.7	259.22	0.71	0.27%
229	POE Isosorbide monoester 10:0	n-10	[M + Na]	5 (x3 Croda, x2 USP)	763.4326	0.0063	763.4456	8.5	267.64	0.29	0.11%
230	POE Isosorbide monoester 10:0	n-11	[M + Na]	5 (x3 Croda, x2 USP)	807.4575	0.0061	807.4718	6.5	275.80	0.29	0.10%
231	POE Isosorbide monoester 10:0	n-12	[M + Na]	5 (x3 Croda, x2 USP)	851.4817	0.0060	851.4980	3.8	283.76	0.41	0.14%
232	POE Isosorbide monoester 10:0	n-13	[M + Na]	5 (x3 Croda, x2 USP)	895.5070	0.0061	895.5242	2.7	291.56	0.40	0.14%
233	POE Isosorbide monoester 10:0	n-14	[M + Na]	5 (x3 Croda, x2 USP)	939.5315	0.0063	939.5504	0.8	299.22	0.40	0.13%
234	POE Isosorbide monoester 10:0	n-15	[M + Na]	5 (x3 Croda, x2 USP)	983.5570	0.0051	983.5766	0.0	306.72	0.19	0.06%
235	POE Isosorbide monoester 10:0	n-16	[M + Na]	5 (x3 Croda, x2 USP)	1027.5825	0.0071	1027.6029	-0.6	314.19	0.23	0.07%
236	POE Isosorbide monoester 10:0	n-17	[M + Na]	5 (x3 Croda, x2 USP)	1071.6067	0.0056	1071.6291	-2.5	321.40	0.19	0.06%
237	POE Isosorbide monoester 12:0	n-5	[M + Na]	5 (x3 Croda, x2 USP)	571.3408	0.0012	571.3458	-5.9	234.11	1.40	0.60%
238	POE Isosorbide monoester 12:0	n-6	[M + Na]	5 (x3 Croda, x2 USP)	615.3685	0.0021	615.3720	-2.4	243.28	0.54	0.22%
239	POE Isosorbide monoester 12:0	n-7	[M + Na]	5 (x3 Croda, x2 USP)	659.3949	0.0021	659.3982	-1.2	250.61	0.41	0.16%
240	POE Isosorbide monoester 12:0	n-8	[M + Na]	5 (x3 Croda, x2 USP)	703.4219	0.0026	703.4244	0.7	258.36	0.50	0.19%
241	POE Isosorbide monoester 12:0	n-9	[M + Na]	5 (x3 Croda, x2 USP)	747.4480	0.0028	747.4507	1.1	266.80	0.30	0.11%
242	POE Isosorbide monoester 12:0	n-10	[M + Na]	5 (x3 Croda, x2 USP)	791.4746	0.0027	791.4769	2.2	274.74	0.37	0.13%
243	POE Isosorbide monoester 12:0	n-11	[M + Na]	5 (x3 Croda, x2 USP)	835.5001	0.0035	835.5031	1.8	282.65	0.28	0.10%
244	POE Isosorbide monoester 12:0	n-12	[M + Na]	5 (x3 Croda, x2 USP)	879.5261	0.0035	879.5293	2.0	290.39	0.25	0.09%
245	POE Isosorbide monoester 12:0	n-13	[M + Na]	5 (x3 Croda, x2 USP)	923.5515	0.0046	923.5555	1.5	297.92	0.29	0.10%
246	POE Isosorbide monoester 12:0	n-14	[M + Na]	5 (x3 Croda, x2 USP)	967.5769	0.0044	967.5817	1.1	305.41	0.28	0.09%
247	POE Isosorbide monoester 12:0	n-15	[M + Na]	5 (x3 Croda, x2 USP)	1011.6020	0.0050	1011.6079	0.5	312.81	0.24	0.08%
248	POE Isosorbide monoester 12:0	n-16	[M + Na]	5 (x3 Croda, x2 USP)	1055.6269	0.0050	1055.6342	-0.3	320.07	0.25	0.08%
249	POE Isosorbide monoester 12:0	n-17	[M + Na]	5 (x3 Croda, x2 USP)	1099.6520	0.0046	1099.6604	-0.9	327.16	0.36	0.11%
250	POE Isosorbide monoester 12:0	n-18	[M + Na]	5 (x3 Croda, x2 USP)	1143.6766	0.0045	1143.6866	-1.8	334.31	0.39	0.12%
251	POE Isosorbide monoester 12:0	n-19	[M + Na]	5 (x3 Croda, x2 USP)	1187.7031	0.0086	1187.7128	-1.2	341.59	0.56	0.17%
252	POE Isosorbide monoester 14:0	n-6	[M + Na]	4 (x2 Croda, x2 USP)	643.3997	0.0038	643.4033	-1.9	252.44	0.52	0.21%
253	POE Isosorbide monoester 14:0	n-7	[M + Na]	5 (x3 Croda, x2 USP)	687.4263	0.0025	687.4295	-0.5	258.95	0.46	0.18%
254	POE Isosorbide monoester 14:0	n-8	[M + Na]	5 (x3 Croda, x2 USP)	731.4531	0.0036	731.4557	0.9	266.16	0.70	0.26%
255	POE Isosorbide monoester 14:0	n-9	[M + Na]	5 (x3 Croda, x2 USP)	775.4788	0.0034	775.4820	0.8	274.15	0.44	0.16%
256	POE Isosorbide monoester 14:0	n-10	[M + Na]	5 (x3 Croda, x2 USP)	819.5047	0.0040	819.5082	1.0	281.62	0.42	0.15%
257	POE Isosorbide monoester 14:0	n-11	[M + Na]	5 (x3 Croda, x2 USP)	863.5303	0.0036	863.5344	0.8	289.29	0.39	0.13%
258	POE Isosorbide monoester 14:0	n-12	[M + Na]	5 (x3 Croda, x2 USP)	907.5555	0.0046	907.5606	0.2	296.87	0.36	0.12%
259	POE Isosorbide monoester 14:0	n-13	[M + Na]	5 (x3 Croda, x2 USP)	951.5812	0.0049	951.5868	0.1	304.30	0.32	0.10%
260	POE Isosorbide monoester 14:0	n-14	[M + Na]	5 (x3 Croda, x2 USP)	995.6059	0.0055	995.6130	-1.0	311.58	0.26	0.08%
261	POE Isosorbide monoester 14:0	n-15	[M + Na]	5 (x3 Croda, x2 USP)	1039.6303	0.0061	1039.6392	-2.2	318.94	0.36	0.11%
262	POE Isosorbide monoester 14:0	n-16	[M + Na]	5 (x3 Croda, x2 USP)	1083.6552	0.0055	1083.6655	-2.8	325.95	0.26	0.08%

= Signal Interference from Neighboring Isotopic Envelope(s)
= Low Signal Abundance and/or Poor Feature Resolution

Table S2. (continued)

	Structural Series	Mono-mer	Ion Form	Measurement Replicates	Avg. *m/z*	StDev *m/z*	Theor. *m/z*	Mass Acc. (ppm)	Avg. *CCS/z* (Å^2/z)	StDev *CCS/z* (Å^2/z)	%RSD *CCS/z* (%)
263	POE Isosorbide monoester 14:0	n-17	[M + Na]	5 (x3 Croda, x2 USP)	1127.6798	0.0063	1127.6917	7.0	333.75	0.93	0.28%
264	POE Isosorbide monoester 14:0	n-18	[M + Na]	5 (x3 Croda, x2 USP)	1171.7052	0.0065	1171.7179	6.1	340.99	1.11	0.33%
265	POE Isosorbide monoester 14:0	n-19	[M + Na]	5 (x3 Croda, x2 USP)	1215.7312	0.0063	1215.7441	5.7	348.04	1.20	0.34%
266	POE Isosorbide monoester 14:0	n-20	[M + Na]	4 (x2 Croda, x2 USP)	1259.7539	0.0088	1259.7703	2.7	353.70	0.54	0.15%
267	POE Isosorbide monoester 14:0	n-21	[M + Na]	5 (x3 Croda, x2 USP)	1303.7778	0.0135	1303.7965	1.0	361.12	1.43	0.40%
268	POE Isosorbide monoester 14:0	n-22	[M + Na]	5 (x3 Croda, x2 USP)	1347.8047	0.0103	1347.8227	1.5	369.26	1.82	0.49%
269	POE Isosorbide monoester 14:0	n-23	[M + Na]	5 (x3 Croda, x2 USP)	1391.8297	0.0119	1391.8489	0.6	375.77	2.55	0.68%
270	POE Isosorbide monoester 14:0	n-24	[M + Na]	5 (x3 Croda, x2 USP)	1435.8552	0.0122	1435.8752	0.1	383.96	2.61	0.68%
271	POE Isosorbide monoester 18:1	n-10	[M + Na]	5 (x3 Croda, x2 USP)	873.5485	0.0012	873.5551	-2.0	292.65	1.18	0.40%
272	POE Isosorbide monoester 18:1	n-11	[M + Na]	5 (x3 Croda, x2 USP)	917.5758	0.0037	917.5813	-0.1	301.06	0.63	0.21%
273	POE Isosorbide monoester 18:1	n-12	[M + Na]	5 (x3 Croda, x2 USP)	961.6012	0.0037	961.6075	-0.5	308.62	2.64	0.85%
274	POE Isosorbide monoester 18:1	n-13	[M + Na]	5 (x3 Croda, x2 USP)	1005.6267	0.0053	1005.6338	-0.7	316.50	1.45	0.46%
275	POE Isosorbide monoester 18:1	n-14	[M + Na]	5 (x3 Croda, x2 USP)	1049.6530	0.0063	1049.6600	-0.1	324.35	0.42	0.13%
276	POE Isosorbide monoester 18:1	n-15	[M + Na]	5 (x3 Croda, x2 USP)	1093.6774	0.0044	1093.6862	-1.4	331.86	0.52	0.16%
277	POE Isosorbide diester 22:0	n-14	[M + Na]	5 (x3 Croda, x2 USP)	1121.6994	0.0083	1121.7175	1.5	340.66	0.78	0.23%
278	POE Isosorbide diester 22:0	n-15	[M + Na]	5 (x3 Croda, x2 USP)	1165.7242	0.0111	1165.7437	0.3	346.16	0.87	0.25%
279	POE Isosorbide diester 22:0	n-16	[M + Na]	5 (x3 Croda, x2 USP)	1209.7487	0.0082	1209.7699	-1.1	352.75	1.03	0.29%
280	POE Isosorbide diester 24:0	n-14	[M + Na]	5 (x3 Croda, x2 USP)	1149.7365	0.0041	1149.7488	6.6	345.85	1.36	0.39%
281	POE Isosorbide diester 24:0	n-15	[M + Na]	5 (x3 Croda, x2 USP)	1193.7618	0.0021	1193.7750	5.5	351.98	0.57	0.16%
282	POE Isosorbide diester 24:0	n-16	[M + Na]	5 (x3 Croda, x2 USP)	1237.7873	0.0085	1237.8012	4.8	358.26	0.38	0.10%
283	POE Isosorbide diester 26:0	n-15	[M + Na]	5 (x3 Croda, x2 USP)	1221.7886	0.0042	1221.8063	1.8	357.43	1.59	0.44%
284	POE Isosorbide diester 26:0	n-16	[M + Na]	5 (x3 Croda, x2 USP)	1265.8151	0.0074	1265.8325	2.0	363.96	0.55	0.15%
285	POE Isosorbide diester 26:0	n-17	[M + Na]	5 (x3 Croda, x2 USP)	1309.8469	0.0061	1309.8587	-1.6	370.43	0.96	0.26%
286	POE Isosorbide diester 26:0	n-18	[M + Na]	5 (x3 Croda, x2 USP)	1353.8640	0.0074	1353.8849	-0.7	375.69	1.16	0.31%
287	POE Isosorbide diester 26:0	n-19	[M + Na]	4 (x3 Croda, x1 USP)	1397.8933	0.0163	1397.9111	1.6	381.38	0.84	0.22%
288	POE Isosorbide diester 28:0	n-12	[M + Na]	5 (x3 Croda, x2 USP)	1117.7494	0.0052	1117.7590	-1.8	343.40	0.72	0.21%
289	POE Isosorbide diester 28:0	n-13	[M + Na]	5 (x3 Croda, x2 USP)	1161.7731	0.0070	1161.7852	6.7	349.92	0.58	0.17%
290	POE Isosorbide diester 28:0	n-14	[M + Na]	5 (x3 Croda, x2 USP)	1205.8024	0.0097	1205.8114	-0.4	356.16	1.47	0.41%
291	POE Isosorbide diester 28:0	n-15	[M + Na]	4 (x2 Croda, x2 USP)	1249.8279	0.0088	1249.8376	-0.6	362.71	1.70	0.47%
292	POE Isosorbide diester 28:0	n-16	[M + Na]	4 (x3 Croda, x1 USP)	1293.8454	0.0083	1293.8638	1.2	367.11	2.26	0.62%
293	POE Isosorbide diester 28:0	n-17	[M + Na]	3 (Croda)	1337.8767	0.0170	1337.8900	-2.5	376.81	0.17	0.05%
294	POE Isosorbide diester 28:0	n-18	[M + Na]	3 (Croda)	1381.8999	0.0056	1381.9162	2.6	380.79	0.35	0.09%
295	POE Isosorbide diester 28:0	n-22	[M + Na]	3 (Croda)	1558.0052	0.0081	1558.0211	2.8	408.15	2.81	0.69%
296	POE Isosorbide diester 28:0	n-23	[M + Na]	3 (Croda)	1602.0310	0.0172	1602.0473	2.4	413.62	2.10	0.51%
297	POE Isosorbide diester 28:0	n-24	[M + Na]	3 (Croda)	1646.0541	0.0119	1646.0735	0.5	420.47	1.18	0.28%
298	POE Isosorbide diester 28:0	n-25	[M + Na]	3 (Croda)	1690.0734	0.0081	1690.0997	-3.5	427.33	1.19	0.28%
299	POE Isosorbide diester 28:0	n-26	[M + Na]	3 (Croda)	1734.1027	0.0105	1734.1259	-1.7	433.85	1.10	0.25%
300	POE Isosorbide diester 28:0	n-27	[M + Na]	3 (Croda)	1778.1224	0.0078	1778.1522	-5.3	439.28	2.36	0.54%
301	POE Isosorbide	n-10	[M + 2Na]	4 (x2 Croda, x2 USP)	316.1493	0.0008	316.1504	-7.4	144.86	0.92	0.64%
302	POE Isosorbide	n-11	[M + 2Na]	5 (x3 Croda, x2 USP)	338.1635	0.0047	338.1634	-2.3	149.02	0.50	0.34%
303	POE Isosorbide	n-12	[M + 2Na]	5 (x3 Croda, x2 USP)	360.1743	0.0010	360.1764	-7.7	154.45	0.26	0.17%
304	POE Isosorbide	n-13	[M + 2Na]	5 (x3 Croda, x2 USP)	382.1874	0.0011	382.1894	-6.3	159.95	0.48	0.30%
305	POE Isosorbide	n-14	[M + 2Na]	5 (x3 Croda, x2 USP)	404.2008	0.0023	404.2024	-4.6	165.06	0.36	0.22%
306	POE Isosorbide	n-15	[M + 2Na]	5 (x3 Croda, x2 USP)	426.2147	0.0044	426.2154	-1.5	170.38	0.27	0.16%
307	POE Isosorbide	n-16	[M + 2Na]	5 (x3 Croda, x2 USP)	448.2283	0.0048	448.2284	0.4	175.45	0.68	0.39%
308	POE Isosorbide	n-17	[M + 2Na]	5 (x3 Croda, x2 USP)	470.2407	0.0020	470.2414	-0.4	180.78	0.48	0.26%
309	POE Isosorbide	n-18	[M + 2Na]	5 (x3 Croda, x2 USP)	492.2556	0.0042	492.2549	2.9	186.07	0.63	0.34%
310	POE Isosorbide	n-19	[M + 2Na]	5 (x3 Croda, x2 USP)	514.2762	0.0210	514.2679	18.1	192.28	1.08	0.56%
311	POE Isosorbide	n-20	[M + 2Na]	2 (USP)	536.2806	0.0016	536.2809	1.7	198.90	0.01	0.01%
312	POE Isosorbide	n-21	[M + 2Na]	2 (USP)	558.2942	0.0039	558.2939	3.1	204.09	1.24	0.61%
313	POE	n-6	[M + Na]	5 (x3 Croda, x2 USP)	305.1555	0.0008	305.1576	-12.2	157.78	0.26	0.17%
314	POE	n-7	[M + Na]	5 (x3 Croda, x2 USP)	349.1824	0.0016	349.1838	-7.2	166.98	0.32	0.19%
315	POE	n-8	[M + Na]	5 (x3 Croda, x2 USP)	393.2091	0.0011	393.2100	-3.8	176.84	0.31	0.17%
316	POE	n-9	[M + Na]	5 (x3 Croda, x2 USP)	437.2353	0.0013	437.2363	-2.5	185.91	0.31	0.17%
317	POE	n-10	[M + Na]	5 (x3 Croda, x2 USP)	481.2610	0.0017	481.2625	-2.4	194.21	0.32	0.16%
318	POE	n-11	[M + Na]	5 (x3 Croda, x2 USP)	525.2871	0.0021	525.2887	-1.8	205.16	0.36	0.18%
319	POE	n-12	[M + Na]	5 (x3 Croda, x2 USP)	569.3137	0.0018	569.3149	-0.1	215.65	0.41	0.19%
320	POE	n-13	[M + Na]	5 (x3 Croda, x2 USP)	613.3392	0.0020	613.3411	-0.7	225.93	0.36	0.16%
321	POE	n-14	[M + Na]	5 (x3 Croda, x2 USP)	657.3653	0.0030	657.3673	-0.3	235.78	0.29	0.12%
322	POE	n-15	[M + Na]	5 (x3 Croda, x2 USP)	701.3917	0.0022	701.3935	0.7	245.21	0.27	0.11%
323	POE	n-16	[M + Na]	5 (x3 Croda, x2 USP)	745.4172	0.0026	745.4197	0.2	254.46	0.25	0.10%
324	POE	n-17	[M + Na]	5 (x3 Croda, x2 USP)	789.4427	0.0022	789.4460	-0.1	263.39	0.26	0.10%
325	POE	n-18	[M + Na]	5 (x3 Croda, x2 USP)	833.4677	0.0039	833.4722	-1.4	272.06	0.37	0.14%
326	POE	n-19	[M + Na]	5 (x3 Croda, x2 USP)	877.4927	0.0045	877.4984	-2.6	280.48	0.48	0.17%
327	POE	n-20	[M + Na]	5 (x3 Croda, x2 USP)	921.5171	0.0044	921.5246	-4.3	288.17	0.54	0.19%
328	POE	n-8	[M + K]	5 (x3 Croda, x2 USP)	409.1816	0.0017	409.1839	-6.0	178.79	0.29	0.16%
329	POE	n-9	[M + K]	5 (x3 Croda, x2 USP)	453.2089	0.0016	453.2099	-1.7	186.65	0.30	0.16%
330	POE	n-10	[M + K]	5 (x3 Croda, x2 USP)	497.2349	0.0017	497.2359	-0.6	196.10	0.33	0.17%

= Signal Interference from Neighboring Isotopic Envelope(s)

= Low Signal Abundance and/or Poor Feature Resolution

Table S2. (continued)

	Structural Series	Mono-mer	Ion Form	Measurement Replicates	Avg. *m/z*	StDev *m/z*	Theor. *m/z*	Mass Acc. (ppm)	Avg. *CCS/z* (Å²/z)	StDev *CCS/z* (Å²/z)	%RSD *CCS/z* (%)
332	POE	n-12	[M + K]	5 (x3 Croda, x2 USP)	585.2872	0.0019	585.2889	0.0	215.02	0.39	0.18%
333	POE	n-13	[M + K]	5 (x3 Croda, x2 USP)	629.3129	0.0019	629.3149	0.2	225.91	0.33	0.15%
334	POE	n-14	[M + K]	5 (x3 Croda, x2 USP)	673.3388	0.0024	673.3409	0.9	235.74	0.27	0.11%
335	POE	n-15	[M + K]	5 (x3 Croda, x2 USP)	717.3655	0.0025	717.3679	1.1	245.90	0.28	0.12%
336	POE	n-16	[M + K]	5 (x3 Croda, x2 USP)	761.3903	0.0022	761.3939	0.0	255.03	0.28	0.11%
337	POE	n-17	[M + K]	5 (x3 Croda, x2 USP)	805.4163	0.0034	805.4199	0.6	264.34	0.28	0.11%
338	POE	n-18	[M + K]	5 (x3 Croda, x2 USP)	849.4413	0.0026	849.4459	0.0	273.27	0.42	0.16%
339	POE monoester 10:0	n-8	[M + Na]	5 (x3 Croda, x2 USP)	547.3386	0.0031	547.3458	22.1	226.24	1.15	0.51%
340	POE monoester 10:0	n-9	[M + Na]	5 (x3 Croda, x2 USP)	591.3599	0.0032	591.3720	12.2	236.13	0.53	0.22%
341	POE monoester 10:0	n-10	[M + Na]	5 (x3 Croda, x2 USP)	635.3843	0.0039	635.3982	8.6	245.06	0.45	0.18%
342	POE monoester 10:0	n-11	[M + Na]	5 (x3 Croda, x2 USP)	679.4074	0.0049	679.4244	3.5	254.13	0.36	0.14%
343	POE monoester 10:0	n-12	[M + Na]	5 (x3 Croda, x2 USP)	723.4322	0.0045	723.4507	1.3	262.74	0.37	0.14%
344	POE monoester 10:0	n-13	[M + Na]	5 (x3 Croda, x2 USP)	767.4577	0.0049	767.4769	0.3	271.22	0.38	0.14%
345	POE monoester 10:0	n-14	[M + Na]	5 (x3 Croda, x2 USP)	811.4828	0.0043	811.5031	-0.9	279.01	0.29	0.10%
346	POE monoester 10:0	n-15	[M + Na]	5 (x3 Croda, x2 USP)	855.5082	0.0045	855.5293	-1.8	287.12	0.22	0.08%
347	POE monoester 10:0	n-16	[M + Na]	5 (x3 Croda, x2 USP)	899.5333	0.0044	899.5555	-3.0	295.20	0.22	0.08%
348	POE monoester 10:0	n-17	[M + Na]	5 (x3 Croda, x2 USP)	943.5583	0.0049	943.5817	-4.0	302.77	0.64	0.21%
349	POE monoester 10:0	n-18	[M + Na]	5 (x3 Croda, x2 USP)	987.5832	0.0040	987.6079	-5.2	310.53	0.21	0.07%
350	POE monoester 12:0	n-6	[M + Na]	5 (x3 Croda, x2 USP)	487.3220	0.0019	487.3247	-4.2	218.35	0.65	0.30%
351	POE monoester 12:0	n-7	[M + Na]	5 (x3 Croda, x2 USP)	531.3481	0.0018	531.3509	-3.1	227.43	0.62	0.27%
352	POE monoester 12:0	n-8	[M + Na]	5 (x3 Croda, x2 USP)	575.3745	0.0024	575.3771	-1.7	236.42	0.40	0.17%
353	POE monoester 12:0	n-9	[M + Na]	5 (x3 Croda, x2 USP)	619.4009	0.0020	619.4033	-0.5	245.13	0.41	0.17%
354	POE monoester 12:0	n-10	[M + Na]	5 (x3 Croda, x2 USP)	663.4264	0.0024	663.4295	-0.8	253.71	0.38	0.15%
355	POE monoester 12:0	n-11	[M + Na]	5 (x3 Croda, x2 USP)	707.4523	0.0026	707.4557	-0.5	261.67	0.31	0.12%
356	POE monoester 12:0	n-12	[M + Na]	5 (x3 Croda, x2 USP)	751.4777	0.0036	751.4820	-0.9	269.76	0.29	0.11%
357	POE monoester 12:0	n-13	[M + Na]	5 (x3 Croda, x2 USP)	795.5032	0.0036	795.5082	-1.1	278.02	0.29	0.10%
358	POE monoester 12:0	n-14	[M + Na]	5 (x3 Croda, x2 USP)	839.5284	0.0040	839.5344	-1.8	285.98	0.24	0.08%
359	POE monoester 12:0	n-15	[M + Na]	5 (x3 Croda, x2 USP)	883.5538	0.0041	883.5606	-2.0	293.82	0.30	0.10%
360	POE monoester 12:0	n-16	[M + Na]	5 (x3 Croda, x2 USP)	927.5785	0.0040	927.5868	-3.1	301.66	0.26	0.09%
361	POE monoester 12:0	n-17	[M + Na]	5 (x3 Croda, x2 USP)	971.6054	0.0038	971.6130	-1.7	309.18	0.26	0.09%
362	POE monoester 12:0	n-18	[M + Na]	5 (x3 Croda, x2 USP)	1015.6270	0.0056	1015.6392	7.3	316.32	0.70	0.22%

(orange) = Signal Interference from Neighboring Isotopic Envelope(s)
(grey) = Low Signal Abundance and/or Poor Feature Resolution

Table S3. Summary of Mass and Mobility Measurements for PS-80

	Structural Series	Mono-mer	Ion Form	Measurement Replicates	Avg. *m/z*	StDev *m/z*	Theor. *m/z*	Mass Acc. (ppm)	Avg. *CCS/z* (Å²/z)	StDev *CCS/z* (Å²/z)	%RSD *CCS/z* (%)
1	POE Sorbitan	n-21	[M + Na]	5 (x3 Croda, x2 USP)	1111.6026	0.008	1111.6090	-0.5	316.24	1.30	0.41%
2	POE Sorbitan	n-22	[M + Na]	5 (x3 Croda, x2 USP)	1155.6295	0.007	1155.6350	0.6	324.13	0.77	0.24%
3	POE Sorbitan	n-23	[M + Na]	5 (x3 Croda, x2 USP)	1199.6561	0.006	1199.6610	1.2	331.90	0.35	0.11%
4	POE Sorbitan	n-24	[M + Na]	5 (x3 Croda, x2 USP)	1243.6811	0.007	1243.6870	0.7	339.92	1.45	0.43%
5	POE Sorbitan	n-25	[M + Na]	5 (x3 Croda, x2 USP)	1287.7074	0.009	1287.7140	0.3	346.12	0.58	0.17%
6	POE Sorbitan	n-26	[M + Na]	5 (x3 Croda, x2 USP)	1331.7345	0.009	1331.7400	1.3	352.07	1.11	0.32%
7	POE Sorbitan	n-27	[M + Na]	5 (x3 Croda, x2 USP)	1375.7611	0.008	1375.7660	1.9	359.25	1.06	0.29%
8	POE Sorbitan	n-28	[M + Na]	5 (x3 Croda, x2 USP)	1419.7886	0.012	1419.7920	3.1	366.31	0.50	0.14%
9	POE Sorbitan	n-29	[M + Na]	5 (x3 Croda, x2 USP)	1463.8111	0.010	1463.8180	0.8	373.44	1.06	0.28%
10	POE Sorbitan	n-30	[M + Na]	5 (x3 Croda, x2 USP)	1507.8373	0.011	1507.8450	0.4	380.25	0.82	0.21%
11	POE Sorbitan	n-17	[M + K]	5 (x3 Croda, x2 USP)	951.4704	0.004	951.4779	-2.8	287.60	0.51	0.18%
12	POE Sorbitan	n-18	[M + K]	5 (x3 Croda, x2 USP)	995.5006	0.004	995.5039	1.8	295.82	1.85	0.62%
13	POE Sorbitan	n-19	[M + K]	5 (x3 Croda, x2 USP)	1039.5287	0.006	1039.5299	4.0	303.41	1.12	0.37%
14	POE Sorbitan	n-20	[M + K]	5 (x3 Croda, x2 USP)	1083.5510	0.007	1083.5569	-0.2	310.89	0.78	0.25%
15	POE Sorbitan	n-21	[M + K]	5 (x3 Croda, x2 USP)	1127.5763	0.009	1127.5829	-0.6	318.50	0.78	0.24%
16	POE Sorbitan	n-22	[M + K]	5 (x3 Croda, x2 USP)	1171.6009	0.007	1171.6089	-1.5	325.91	1.10	0.34%
17	POE Sorbitan	n-23	[M + K]	5 (x3 Croda, x2 USP)	1215.6308	0.007	1215.6349	1.9	332.87	0.49	0.15%
18	POE Sorbitan	n-24	[M + K]	5 (x3 Croda, x2 USP)	1259.6550	0.009	1259.6609	0.7	340.73	0.95	0.28%
19	POE Sorbitan	n-25	[M + K]	5 (x3 Croda, x2 USP)	1303.6821	0.009	1303.6879	0.9	347.19	0.90	0.26%
20	POE Sorbitan	n-26	[M + K]	5 (x3 Croda, x2 USP)	1347.7078	0.009	1347.7139	0.9	353.80	0.92	0.26%
21	POE Sorbitan	n-27	[M + K]	5 (x3 Croda, x2 USP)	1391.7341	0.010	1391.7399	1.3	360.79	0.98	0.27%
22	POE Sorbitan	n-28	[M + K]	4 (x2 Croda, x2 USP)	1435.7583	0.009	1435.7659	0.1	367.69	1.59	0.43%
23	POE Sorbitan monoester 18:0	n-6	[M + Na]	5 (x3 Croda, x2 USP)	717.4547	0.004	717.4765	-1.0	264.61	0.25	0.09%
24	POE Sorbitan monoester 18:0	n-7	[M + Na]	5 (x3 Croda, x2 USP)	761.4813	0.004	761.5027	-0.4	273.34	0.14	0.05%
25	POE Sorbitan monoester 18:0	n-8	[M + Na]	5 (x3 Croda, x2 USP)	805.5071	0.004	805.5289	-1.0	281.60	0.11	0.04%
26	POE Sorbitan monoester 18:0	n-9	[M + Na]	5 (x3 Croda, x2 USP)	849.5333	0.004	849.5551	-1.0	289.67	0.11	0.04%
27	POE Sorbitan monoester 18:0	n-10	[M + Na]	5 (x3 Croda, x2 USP)	893.5598	0.005	893.5813	-0.7	296.86	0.16	0.05%
28	POE Sorbitan monoester 18:0	n-11	[M + Na]	5 (x3 Croda, x2 USP)	937.5856	0.005	937.6075	-1.1	304.57	0.13	0.04%
29	POE Sorbitan monoester 18:0	n-12	[M + Na]	5 (x3 Croda, x2 USP)	981.6113	0.005	981.6338	-1.6	312.34	0.19	0.06%
30	POE Sorbitan monoester 18:0	n-13	[M + Na]	5 (x3 Croda, x2 USP)	1025.6372	0.006	1025.6600	-1.8	319.81	0.26	0.08%
31	POE Sorbitan monoester 18:0	n-14	[M + Na]	5 (x3 Croda, x2 USP)	1069.6629	0.006	1069.6862	-2.3	327.14	0.36	0.11%
32	POE Sorbitan monoester 18:0	n-15	[M + Na]	5 (x3 Croda, x2 USP)	1113.6877	0.007	1113.7124	-3.6	334.13	0.23	0.07%
33	POE Sorbitan monoester 18:0	n-16	[M + Na]	5 (x3 Croda, x2 USP)	1157.7128	0.007	1157.7386	-4.4	340.85	0.37	0.11%
34	POE Sorbitan monoester 18:0	n-17	[M + Na]	5 (x3 Croda, x2 USP)	1201.7413	0.009	1201.7648	-2.4	348.56	1.62	0.46%
35	POE Sorbitan monoester 18:0	n-18	[M + Na]	5 (x3 Croda, x2 USP)	1245.7658	0.008	1245.7910	-3.7	354.31	0.98	0.28%
36	POE Sorbitan monoester 18:1	n-10	[M + Na]	5 (x3 Croda, x2 USP)	891.5500	0.006	891.5657	5.9	295.35	0.17	0.06%
37	POE Sorbitan monoester 18:1	n-11	[M + Na]	5 (x3 Croda, x2 USP)	935.5753	0.005	935.5919	4.6	302.65	0.21	0.07%
38	POE Sorbitan monoester 18:1	n-12	[M + Na]	5 (x3 Croda, x2 USP)	979.5999	0.006	979.6181	2.8	310.13	0.33	0.11%
39	POE Sorbitan monoester 18:1	n-13	[M + Na]	5 (x3 Croda, x2 USP)	1023.6245	0.006	1023.6443	1.0	317.25	0.47	0.15%
40	POE Sorbitan monoester 18:1	n-14	[M + Na]	5 (x3 Croda, x2 USP)	1067.6502	0.007	1067.6705	0.4	324.37	0.48	0.15%
41	POE Sorbitan monoester 18:1	n-15	[M + Na]	5 (x3 Croda, x2 USP)	1111.6737	0.005	1111.6967	-2.0	331.43	0.80	0.24%
42	POE Sorbitan monoester 18:1	n-16	[M + Na]	5 (x3 Croda, x2 USP)	1155.6987	0.005	1155.7230	-3.1	337.86	0.59	0.17%
43	POE Sorbitan monoester 18:1	n-17	[M + Na]	5 (x3 Croda, x2 USP)	1199.7251	0.007	1199.7492	-2.8	344.71	0.41	0.12%
44	POE Sorbitan monoester 18:1	n-18	[M + Na]	5 (x3 Croda, x2 USP)	1243.7563	0.006	1243.7754	1.3	351.37	1.07	0.30%
45	POE Sorbitan monoester 18:1	n-19	[M + Na]	5 (x3 Croda, x2 USP)	1287.7871	0.007	1287.8016	4.7	358.50	0.59	0.16%
46	POE Sorbitan monoester 18:1	n-20	[M + Na]	5 (x3 Croda, x2 USP)	1331.8174	0.008	1331.8278	-2.4	365.79	0.73	0.20%
47	POE Sorbitan monoester 18:1	n-21	[M + Na]	5 (x3 Croda, x2 USP)	1375.8474	0.011	1375.8540	0.7	372.17	1.09	0.29%
48	POE Sorbitan monoester 18:1	n-22	[M + Na]	5 (x3 Croda, x2 USP)	1419.8725	0.009	1419.8802	0.0	378.89	1.08	0.29%
49	POE Sorbitan monoester 18:1	n-23	[M + Na]	5 (x3 Croda, x2 USP)	1463.8999	0.009	1463.9065	1.0	384.91	0.92	0.24%
50	POE Sorbitan monoester 18:1	n-24	[M + Na]	5 (x3 Croda, x2 USP)	1507.9249	0.009	1507.9327	0.4	391.20	0.62	0.16%
51	POE Sorbitan monoester 18:1	n-25	[M + Na]	5 (x3 Croda, x2 USP)	1551.9509	0.010	1551.9589	0.4	397.35	0.88	0.22%
52	POE Sorbitan monoester 18:1	n-26	[M + Na]	5 (x3 Croda, x2 USP)	1595.9781	0.009	1595.9851	1.2	403.79	0.88	0.22%
53	POE Sorbitan monoester 18:1	n-27	[M + Na]	5 (x3 Croda, x2 USP)	1640.0038	0.010	1640.0113	1.0	410.55	1.02	0.25%
54	POE Sorbitan monoester 18:1	n-28	[M + Na]	5 (x3 Croda, x2 USP)	1684.0458	0.037	1684.0375	10.5	415.86	1.60	0.38%
55	POE Sorbitan monoester 18:1	n-29	[M + Na]	5 (x3 Croda, x2 USP)	1728.0518	0.013	1728.0637	-1.3	421.26	2.30	0.55%
56	POE Sorbitan monoester 18:1	n-30	[M + Na]	5 (x3 Croda, x2 USP)	1772.0814	0.010	1772.0899	0.8	428.46	1.92	0.45%
57	POE Sorbitan monoester 18:1	n-31	[M + Na]	3 (Croda)	1816.1088	0.014	1816.1162	1.6	434.51	1.10	0.25%
58	POE Sorbitan monoester 18:2	n-9	[M + Na]	5 (x3 Croda, x2 USP)	845.5020	0.005	845.5238	-20.7	286.49	0.19	0.07%
59	POE Sorbitan monoester 18:2	n-10	[M + Na]	5 (x3 Croda, x2 USP)	889.5280	0.005	889.5500	-19.7	293.83	0.15	0.05%
60	POE Sorbitan monoester 18:2	n-11	[M + Na]	5 (x3 Croda, x2 USP)	933.5539	0.005	933.5762	-18.8	301.22	0.29	0.10%
61	POE Sorbitan monoester 18:2	n-12	[M + Na]	5 (x3 Croda, x2 USP)	977.5800	0.006	977.6025	-17.8	308.45	0.27	0.09%
62	POE Sorbitan monoester 18:2	n-13	[M + Na]	5 (x3 Croda, x2 USP)	1021.6057	0.006	1021.6287	-17.2	315.92	0.36	0.12%
63	POE Sorbitan monoester 18:2	n-14	[M + Na]	5 (x3 Croda, x2 USP)	1065.6324	0.006	1065.6549	-15.8	322.91	0.33	0.10%
64	POE Sorbitan monoester 18:2	n-15	[M + Na]	5 (x3 Croda, x2 USP)	1109.6574	0.006	1109.6811	-16.0	330.34	0.52	0.16%
65	POE Sorbitan monoester 18:2	n-16	[M + Na]	5 (x3 Croda, x2 USP)	1153.6829	0.007	1153.7073	-15.8	337.09	0.59	0.18%
66	POE Sorbitan monoester 18:2	n-17	[M + Na]	5 (x3 Croda, x2 USP)	1197.7095	0.008	1197.7335	-14.7	344.06	0.58	0.17%
67	POE Sorbitan monoester 18:2	n-18	[M + Na]	5 (x3 Croda, x2 USP)	1241.7349	0.008	1241.7597	-3.4	350.90	0.64	0.18%
68	POE Sorbitan monoester 18:2	n-19	[M + Na]	5 (x3 Croda, x2 USP)	1285.7618	0.009	1285.7859	-2.7	357.86	0.93	0.26%

= Signal Interference from Neighboring Isotopic Envelope(s)
= Low Signal Abundance and/or Poor Feature Resolution

Table S3. (continued)

	Structural Series	Monomer	Ion Form	Measurement Replicates	Avg. *m/z*	StDev *m/z*	Theor. *m/z*	Mass Acc. (ppm)	Avg. *CCS/z* ($Å^2/z$)	StDev *CCS/z* ($Å^2/z$)	%RSD *CCS/z* (%)
69	POE Sorbitan diester 36:2	n-20	[M + Na]	5 (x3 Croda, x2 USP)	1596.0462	0.025	1596.0731	2.8	414.26	1.71	0.41%
70	POE Sorbitan diester 36:2	n-21	[M + Na]	5 (x3 Croda, x2 USP)	1640.0759	0.025	1640.0993	4.8	419.30	1.10	0.26%
71	POE Sorbitan diester 36:2	n-22	[M + Na]	5 (x3 Croda, x2 USP)	1684.1012	0.015	1684.1255	1.4	425.39	1.84	0.43%
72	POE Sorbitan diester 36:2	n-23	[M + Na]	5 (x3 Croda, x2 USP)	1728.1314	0.006	1728.1518	1.0	433.03	1.34	0.31%
73	POE Sorbitan diester 36:2	n-24	[M + Na]	4 (x3 Croda, x1 USP)	1772.1655	0.008	1772.1780	-1.4	437.58	1.64	0.37%
74	POE Sorbitan diester 36:2	n-25	[M + Na]	4 (x3 Croda, x1 USP)	1816.1936	0.005	1816.2042	-0.2	444.80	0.95	0.21%
75	POE Sorbitan diester 36:2	n-26	[M + Na]	3 (Croda)	1860.2206	0.010	1860.2304	0.4	449.73	0.69	0.15%
76	POE Isosorbide	n-7	[M + Na]	5 (x3 Croda, x2 USP)	477.2296	0.003	477.2310	1.1	195.16	0.20	0.10%
77	POE Isosorbide	n-8	[M + Na]	5 (x3 Croda, x2 USP)	521.2562	0.002	521.2570	2.6	204.46	0.22	0.11%
78	POE Isosorbide	n-9	[M + Na]	5 (x3 Croda, x2 USP)	565.2832	0.003	565.2840	3.0	213.15	0.11	0.05%
79	POE Isosorbide	n-10	[M + Na]	5 (x3 Croda, x2 USP)	609.3088	0.003	609.3100	2.5	221.63	0.17	0.08%
80	POE Isosorbide	n-11	[M + Na]	5 (x3 Croda, x2 USP)	653.3352	0.003	653.3360	3.4	230.66	0.16	0.07%
81	POE Isosorbide	n-12	[M + Na]	5 (x3 Croda, x2 USP)	697.3614	0.004	697.3620	3.9	240.07	0.16	0.07%
82	POE Isosorbide	n-13	[M + Na]	5 (x3 Croda, x2 USP)	741.3873	0.004	741.3880	3.9	249.43	0.14	0.06%
83	POE Isosorbide	n-14	[M + Na]	5 (x3 Croda, x2 USP)	785.4135	0.004	785.4150	3.1	258.64	0.17	0.07%
84	POE Isosorbide	n-15	[M + Na]	5 (x3 Croda, x2 USP)	829.4393	0.004	829.4410	3.0	267.52	0.14	0.05%
85	POE Isosorbide	n-16	[M + Na]	5 (x3 Croda, x2 USP)	873.4652	0.004	873.4670	3.0	275.78	0.31	0.11%
86	POE Isosorbide	n-17	[M + Na]	5 (x3 Croda, x2 USP)	917.4904	0.005	917.4930	2.2	284.09	0.33	0.12%
87	POE Isosorbide	n-18	[M + Na]	5 (x3 Croda, x2 USP)	961.5156	0.004	961.5200	0.5	292.40	0.43	0.15%
88	POE Isosorbide	n-19	[M + Na]	5 (x3 Croda, x2 USP)	1005.5414	0.005	1005.5460	0.7	300.13	0.72	0.24%
89	POE Isosorbide	n-20	[M + Na]	5 (x3 Croda, x2 USP)	1049.5664	0.006	1049.5720	-0.1	307.44	0.95	0.31%
90	POE Isosorbide	n-21	[M + Na]	5 (x3 Croda, x2 USP)	1093.5930	0.009	1093.5980	0.7	315.51	0.97	0.31%
91	POE Isosorbide	n-22	[M + Na]	5 (x3 Croda, x2 USP)	1137.6168	0.008	1137.6240	-1.1	321.52	1.45	0.45%
92	POE Isosorbide	n-23	[M + Na]	5 (x3 Croda, x2 USP)	1181.6423	0.008	1181.6510	-2.0	331.07	1.15	0.35%
93	POE Isosorbide	n-24	[M + Na]	1 (Croda)	1225.6758	--	1225.6770	4.4	337.18	--	--
94	POE Isosorbide	n-9	[M + K]	5 (x3 Croda, x2 USP)	581.2554	0.003	581.2579	0.1	215.08	0.59	0.28%
95	POE Isosorbide	n-10	[M + K]	5 (x3 Croda, x2 USP)	625.2819	0.003	625.2839	1.3	223.40	0.35	0.16%
96	POE Isosorbide	n-11	[M + K]	5 (x3 Croda, x2 USP)	669.3088	0.003	669.3099	2.9	232.08	0.15	0.07%
97	POE Isosorbide	n-12	[M + K]	5 (x3 Croda, x2 USP)	713.3350	0.004	713.3359	3.5	241.04	0.15	0.06%
98	POE Isosorbide	n-13	[M + K]	5 (x3 Croda, x2 USP)	757.3610	0.004	757.3619	3.6	250.32	0.18	0.07%
99	POE Isosorbide	n-14	[M + K]	5 (x3 Croda, x2 USP)	801.3869	0.004	801.3889	2.3	259.38	0.21	0.08%
100	POE Isosorbide	n-15	[M + K]	5 (x3 Croda, x2 USP)	845.4129	0.005	845.4149	2.6	268.39	0.34	0.13%
101	POE Isosorbide	n-16	[M + K]	5 (x3 Croda, x2 USP)	889.4383	0.005	889.4409	2.1	277.18	0.45	0.16%
102	POE Isosorbide	n-17	[M + K]	5 (x3 Croda, x2 USP)	933.4637	0.006	933.4669	1.7	285.53	0.17	0.06%
103	POE Isosorbide	n-18	[M + K]	5 (x3 Croda, x2 USP)	977.4890	0.006	977.4939	0.1	293.37	0.58	0.20%
104	POE Isosorbide	n-19	[M + K]	5 (x3 Croda, x2 USP)	1021.5145	0.005	1021.5199	-0.1	301.90	0.67	0.22%
105	POE Isosorbide monoester 14:0	n-10	[M + Na]	5 (x3 Croda, x2 USP)	819.4940	0.014	819.5082	8.3	283.75	1.38	0.49%
106	POE Isosorbide monoester 14:0	n-11	[M + Na]	5 (x3 Croda, x2 USP)	863.5186	0.011	863.5344	6.0	289.83	0.95	0.33%
107	POE Isosorbide monoester 14:0	n-12	[M + Na]	5 (x3 Croda, x2 USP)	907.5417	0.011	907.5606	2.3	296.93	0.39	0.13%
108	POE Isosorbide monoester 14:0	n-13	[M + Na]	5 (x3 Croda, x2 USP)	951.5691	0.010	951.5868	3.4	304.65	0.86	0.28%
109	POE Isosorbide monoester 14:0	n-14	[M + Na]	5 (x3 Croda, x2 USP)	995.5916	0.008	995.6130	-0.5	311.83	0.44	0.14%
110	POE Isosorbide monoester 14:0	n-15	[M + Na]	5 (x3 Croda, x2 USP)	1039.6192	0.009	1039.6392	0.8	318.99	0.55	0.17%
111	POE Isosorbide monoester 14:0	n-16	[M + Na]	5 (x3 Croda, x2 USP)	1083.6428	0.007	1083.6655	-1.7	326.08	0.24	0.07%
112	POE Isosorbide monoester 14:0	n-17	[M + Na]	5 (x3 Croda, x2 USP)	1127.6681	0.009	1127.6917	-2.5	333.77	1.00	0.30%
113	POE Isosorbide monoester 14:0	n-18	[M + Na]	5 (x3 Croda, x2 USP)	1171.6928	0.008	1171.7179	-3.7	340.48	1.02	0.30%
114	POE Isosorbide monoester 14:0	n-19	[M + Na]	5 (x3 Croda, x2 USP)	1215.7218	0.007	1215.7441	-1.4	347.51	1.83	0.53%
115	POE Isosorbide monoester 18:1	n-8	[M + Na]	5 (x3 Croda, x2 USP)	785.5001	0.004	785.5027	1.6	277.57	0.57	0.21%
116	POE Isosorbide monoester 18:1	n-9	[M + Na]	5 (x3 Croda, x2 USP)	829.5267	0.005	829.5289	2.3	285.36	0.48	0.17%
117	POE Isosorbide monoester 18:1	n-10	[M + Na]	5 (x3 Croda, x2 USP)	873.5533	0.005	873.5551	3.0	292.35	0.27	0.09%
118	POE Isosorbide monoester 18:1	n-11	[M + Na]	5 (x3 Croda, x2 USP)	917.5799	0.005	917.5813	3.6	299.70	0.21	0.07%
119	POE Isosorbide monoester 18:1	n-12	[M + Na]	5 (x3 Croda, x2 USP)	961.6061	0.006	961.6075	3.6	307.36	0.23	0.07%
120	POE Isosorbide monoester 18:1	n-13	[M + Na]	5 (x3 Croda, x2 USP)	1005.6317	0.006	1005.6338	3.1	314.62	0.32	0.10%
121	POE Isosorbide monoester 18:1	n-14	[M + Na]	5 (x3 Croda, x2 USP)	1049.6585	0.006	1049.6600	3.8	322.02	0.36	0.11%
122	POE Isosorbide monoester 18:1	n-15	[M + Na]	5 (x3 Croda, x2 USP)	1093.6837	0.007	1093.6862	3.0	328.99	0.45	0.14%
123	POE Isosorbide monoester 18:1	n-16	[M + Na]	5 (x3 Croda, x2 USP)	1137.7091	0.007	1137.7124	2.4	336.08	0.47	0.14%
124	POE Isosorbide monoester 18:1	n-17	[M + Na]	5 (x3 Croda, x2 USP)	1181.7349	0.008	1181.7386	2.2	343.16	0.62	0.18%
125	POE Isosorbide monoester 18:1	n-18	[M + Na]	5 (x3 Croda, x2 USP)	1225.7595	0.008	1225.7648	1.0	349.71	0.51	0.15%
126	POE	n-8	[M + Na]	5 (x3 Croda, x2 USP)	393.2095	0.002	393.2100	2.3	176.87	0.39	0.22%
127	POE	n-9	[M + Na]	5 (x3 Croda, x2 USP)	437.2360	0.002	437.2360	3.9	185.93	0.29	0.16%
128	POE	n-10	[M + Na]	5 (x3 Croda, x2 USP)	481.2607	0.002	481.2620	1.5	194.16	0.26	0.13%
129	POE	n-11	[M + Na]	5 (x3 Croda, x2 USP)	525.2876	0.003	525.2890	1.6	205.07	0.18	0.09%
130	POE	n-12	[M + Na]	5 (x3 Croda, x2 USP)	569.3144	0.003	569.3150	3.4	215.43	0.20	0.09%
131	POE	n-13	[M + Na]	5 (x3 Croda, x2 USP)	613.3399	0.003	613.3410	2.8	225.57	0.22	0.10%
132	POE	n-14	[M + Na]	5 (x3 Croda, x2 USP)	657.3660	0.003	657.3670	3.1	235.38	0.17	0.07%
133	POE	n-15	[M + Na]	5 (x3 Croda, x2 USP)	701.3925	0.003	701.3940	2.6	244.81	0.18	0.08%
134	POE	n-16	[M + Na]	5 (x3 Croda, x2 USP)	745.4182	0.004	745.4200	2.4	254.13	0.09	0.04%
135	POE	n-17	[M + Na]	5 (x3 Croda, x2 USP)	789.4444	0.003	789.4460	2.9	263.16	0.15	0.06%

= Signal Interference from Neighboring Isotopic Envelope(s)
= Low Signal Abundance and/or Poor Feature Resolution

Table S3. (continued)

	Structural Series	Mono-mer	Ion Form	Measurement Replicates	Avg. *m/z*	StDev *m/z*	Theor. *m/z*	Mass Acc. (ppm)	Avg. *CCS/z* (Å²/z)	StDev *CCS/z* (Å²/z)	%RSD *CCS/z* (%)
136	POE	n-18	[M + Na]	5 (x3 Croda, x2 USP)	833.4699	0.005	833.4720	2.5	271.93	0.24	0.09%
137	POE	n-19	[M + Na]	5 (x3 Croda, x2 USP)	877.4944	0.004	877.4980	0.9	280.06	0.46	0.16%
138	POE	n-20	[M + Na]	5 (x3 Croda, x2 USP)	921.5202	0.005	921.5250	-0.2	288.11	0.45	0.16%
139	POE	n-8	[M + K]	5 (x3 Croda, x2 USP)	409.1821	0.002	409.1839	-0.7	178.74	0.45	0.25%
140	POE	n-9	[M + K]	5 (x3 Croda, x2 USP)	453.2098	0.002	453.2099	3.6	186.69	0.29	0.16%
141	POE	n-10	[M + K]	5 (x3 Croda, x2 USP)	497.2357	0.003	497.2359	3.6	196.06	0.24	0.12%
142	POE	n-11	[M + K]	5 (x3 Croda, x2 USP)	541.2615	0.003	541.2629	1.7	205.35	0.22	0.11%
143	POE	n-12	[M + K]	5 (x3 Croda, x2 USP)	585.2883	0.003	585.2889	3.3	214.83	0.26	0.12%
144	POE	n-13	[M + K]	5 (x3 Croda, x2 USP)	629.3139	0.003	629.3149	2.9	225.57	0.16	0.07%
145	POE	n-14	[M + K]	5 (x3 Croda, x2 USP)	673.3399	0.004	673.3409	3.2	235.30	0.20	0.08%
146	POE	n-15	[M + K]	5 (x3 Croda, x2 USP)	717.3668	0.004	717.3679	3.2	245.42	0.15	0.06%
147	POE	n-16	[M + K]	5 (x3 Croda, x2 USP)	761.3921	0.004	761.3939	2.4	254.76	0.14	0.05%
148	POE	n-17	[M + K]	5 (x3 Croda, x2 USP)	805.4184	0.004	805.4199	3.1	263.98	0.17	0.06%
149	POE	n-18	[M + K]	5 (x3 Croda, x2 USP)	849.4436	0.004	849.4459	2.2	272.87	0.35	0.13%
150	POE	n-19	[M + K]	5 (x3 Croda, x2 USP)	893.4688	0.005	893.4719	1.6	281.27	0.40	0.14%
151	POE	n-20	[M + K]	5 (x3 Croda, x2 USP)	937.4943	0.005	937.4989	0.2	289.47	0.28	0.10%
152	POE monoester 14:0	n-10	[M + Na]	5 (x3 Croda, x2 USP)	691.4421	0.009	691.4608	3.5	261.77	0.48	0.18%
153	POE monoester 14:0	n-11	[M + Na]	5 (x3 Croda, x2 USP)	735.4677	0.008	735.4870	2.3	269.16	0.70	0.26%
154	POE monoester 14:0	n-12	[M + Na]	5 (x3 Croda, x2 USP)	779.4923	0.005	779.5133	0.1	277.36	0.52	0.19%
155	POE monoester 14:0	n-13	[M + Na]	5 (x3 Croda, x2 USP)	823.5185	0.006	823.5395	0.0	285.03	0.60	0.21%
156	POE monoester 14:0	n-14	[M + Na]	5 (x3 Croda, x2 USP)	867.5449	0.006	867.5657	0.2	292.60	0.26	0.09%
157	POE monoester 14:0	n-15	[M + Na]	5 (x3 Croda, x2 USP)	911.5712	0.008	911.5919	0.2	300.20	0.30	0.10%
158	POE monoester 14:0	n-16	[M + Na]	5 (x3 Croda, x2 USP)	955.5976	0.006	955.6181	0.5	308.38	1.08	0.35%
159	POE monoester 14:0	n-17	[M + Na]	5 (x3 Croda, x2 USP)	999.6248	0.008	999.6443	1.3	315.46	0.30	0.09%
160	POE monoester 18:1	n-5	[M + Na]	5 (x3 Croda, x2 USP)	525.3735	0.002	525.3767	-1.8	231.61	0.97	0.42%
161	POE monoester 18:1	n-6	[M + Na]	5 (x3 Croda, x2 USP)	569.3977	0.007	569.4029	-4.7	239.90	1.41	0.59%
162	POE monoester 18:1	n-7	[M + Na]	5 (x3 Croda, x2 USP)	613.4269	0.003	613.4291	0.9	247.78	0.29	0.12%
163	POE monoester 18:1	n-8	[M + Na]	5 (x3 Croda, x2 USP)	657.4525	0.004	657.4553	0.3	256.28	0.25	0.10%
164	POE monoester 18:1	n-9	[M + Na]	5 (x3 Croda, x2 USP)	701.4793	0.003	701.4816	1.6	264.50	0.15	0.06%
165	POE monoester 18:1	n-10	[M + Na]	5 (x3 Croda, x2 USP)	745.5059	0.004	745.5078	2.4	272.67	0.24	0.09%
166	POE monoester 18:1	n-11	[M + Na]	5 (x3 Croda, x2 USP)	789.5321	0.004	789.5340	2.5	280.29	0.15	0.05%
167	POE monoester 18:1	n-12	[M + Na]	5 (x3 Croda, x2 USP)	833.5580	0.004	833.5602	2.3	287.73	0.12	0.04%
168	POE monoester 18:1	n-13	[M + Na]	5 (x3 Croda, x2 USP)	877.5844	0.004	877.5864	2.7	295.76	0.09	0.03%
169	POE monoester 18:1	n-14	[M + Na]	5 (x3 Croda, x2 USP)	921.6106	0.005	921.6126	2.9	303.42	0.23	0.08%
170	POE monoester 18:1	n-15	[M + Na]	5 (x3 Croda, x2 USP)	965.6364	0.005	965.6388	2.7	311.06	0.22	0.07%
171	POE monoester 18:1	n-16	[M + Na]	5 (x3 Croda, x2 USP)	1009.6621	0.006	1009.6651	2.2	318.18	1.06	0.33%
172	POE monoester 18:1	n-17	[M + Na]	5 (x3 Croda, x2 USP)	1053.6875	0.006	1053.6913	1.7	325.91	0.40	0.12%
173	POE monoester 18:1	n-18	[M + Na]	5 (x3 Croda, x2 USP)	1097.7125	0.006	1097.7175	0.8	332.96	0.59	0.18%
174	POE diester 34:2	n-10	[M + Na]	5 (x3 Croda, x2 USP)	981.7026	0.008	981.7218	1.7	325.33	1.36	0.42%
175	POE diester 34:2	n-11	[M + Na]	5 (x3 Croda, x2 USP)	1025.7250	0.006	1025.7480	-2.1	332.49	1.53	0.46%
176	POE diester 34:2	n-12	[M + Na]	5 (x3 Croda, x2 USP)	1069.7501	0.005	1069.7742	-3.1	339.45	0.90	0.26%
177	POE diester 34:2	n-13	[M + Na]	5 (x3 Croda, x2 USP)	1113.7758	0.006	1113.8004	-3.5	346.36	0.77	0.22%
178	POE diester 36:2	n-7	[M + Na]	5 (x3 Croda, x2 USP)	877.6666	0.004	877.6744	-3.9	307.96	1.22	0.40%
179	POE diester 36:2	n-8	[M + Na]	5 (x3 Croda, x2 USP)	921.6939	0.008	921.7007	-2.2	317.07	0.64	0.20%
180	POE diester 36:2	n-9	[M + Na]	5 (x3 Croda, x2 USP)	965.7223	0.007	965.7269	0.5	325.94	1.59	0.49%
181	POE diester 36:2	n-10	[M + Na]	5 (x3 Croda, x2 USP)	1009.7462	0.005	1009.7531	-1.6	332.16	0.86	0.26%
182	POE diester 36:2	n-11	[M + Na]	5 (x3 Croda, x2 USP)	1053.7740	0.006	1053.7793	0.2	338.37	1.08	0.32%
183	POE diester 36:2	n-12	[M + Na]	5 (x3 Croda, x2 USP)	1097.7994	0.006	1097.8055	-0.3	344.84	0.57	0.17%
184	POE monoester 18:1	n-13	[M + 2Na]	5 (x3 Croda, x2 USP)	450.2843	0.002	450.2881	-4.6	177.80	0.53	0.30%
185	POE monoester 18:1	n-14	[M + 2Na]	5 (x3 Croda, x2 USP)	472.2986	0.003	472.3012	-1.4	183.05	0.49	0.27%
186	POE monoester 18:1	n-15	[M + 2Na]	5 (x3 Croda, x2 USP)	494.3111	0.002	494.3143	-2.4	189.23	0.44	0.23%
187	POE monoester 18:1	n-16	[M + 2Na]	5 (x3 Croda, x2 USP)	516.3262	0.006	516.3274	1.9	194.66	1.41	0.72%
188	POE monoester 18:1	n-17	[M + 2Na]	5 (x3 Croda, x2 USP)	538.3379	0.005	538.3405	-0.6	199.38	1.07	0.54%
189	POE monoester 18:1	n-18	[M + 2Na]	5 (x3 Croda, x2 USP)	560.3498	0.003	560.3536	-2.4	205.74	1.12	0.54%

= Signal Interference from Neighboring Isotopic Envelope(s)
= Low Signal Abundance and/or Poor Feature Resolution

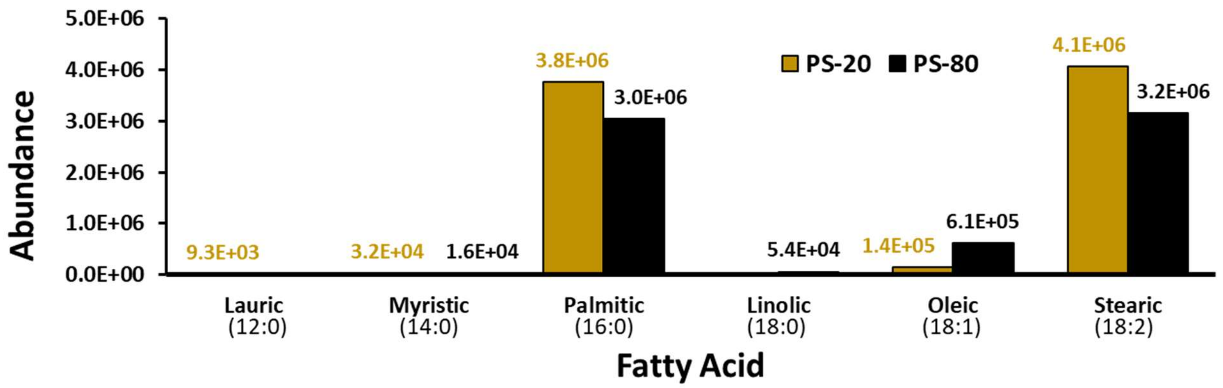

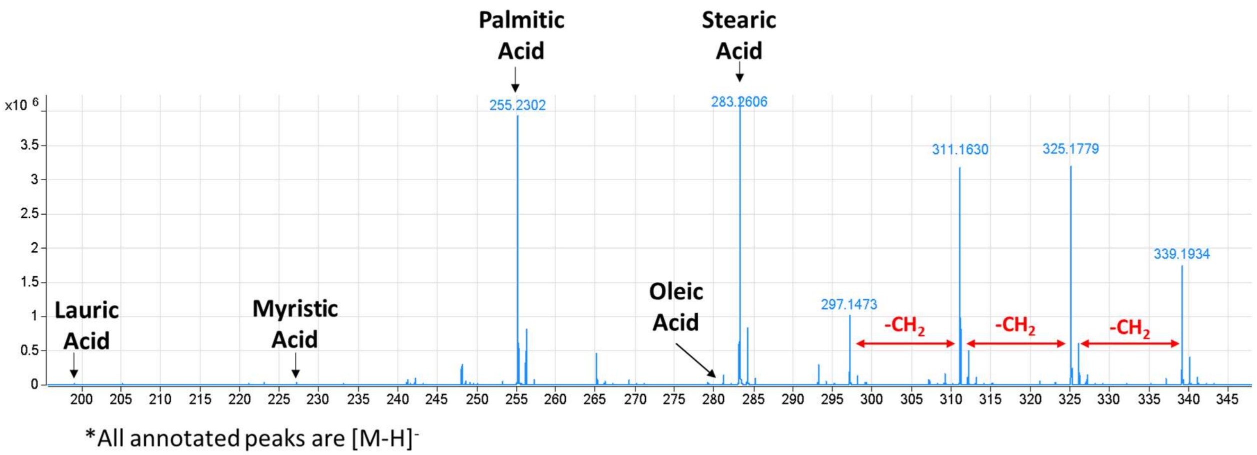

Figure S3. Negative Mode Summary of PS-20 and PS-80 (USP)

Table S4. Details of Linear Mobility-Mass Correlation Fits for PS-20 & PS-80

PS-20

Series	Monomers	Points in Series	*m/z* Range	Adduct	Linear Fit Equation	R^2	Power Fit Equation	R^2
POE Sorbitan	n-17 to n-30	14	935-1508	[M + Na]	y = 0.1630x + 135.43	R^2 = 0.9991	y= 5.0003x^0.5917	R^2 = 0.9999
POE Sorbitan	n-16 to n-27	12	907-1392	[M + K]	y = 0.1682x + 128.08	R^2 = 0.9994	y= 4.7458x^0.5985	R^2 = 0.9999
POE Sorbitan monoester 8:0	n-17 to n-26	10	1061-1458	[M + Na]	y = 0.1577x + 150.00	R^2 = 0.9998	y= 6.1154x^0.5666	R^2 = 0.9991
POE Sorbitan monoester 10:0	n-16 to n-30	15	1045-1662	[M + Na]	y = 0.1529x + 157.27	R^2 = 0.9994	y= 6.2476x^0.5642	R^2 = 0.9998
POE Sorbitan monoester 12:0	n-15 to n-31	17	1029-1735	[M + Na]	y = 0.1512x + 160.49	R^2 = 0.9991	y= 6.4105x^0.5611	R^2 = 0.9999
POE Sorbitan monoester 14:0	n-19 to n-29	11	1233-1674	[M + Na]	y = 0.1481x + 166.68	R^2 = 0.9996	y= 6.3951x^0.5618	R^2 = 0.9997
POE Sorbitan monoester 18:1	n-7 to n-29	23	759-1729	[M + Na]	y = 0.1587x + 154.85	R^2 = 0.9992	y= 7.1936x^0.5470	R^2 = 0.9992
POE Sorbitan diester 22:0	n-15 to n-28	14	1271-1757	[M + Na]	y = 0.1517x + 166.83	R^2 = 0.9975	y= 6.0674x^0.5710	R^2 = 0.9991
POE Sorbitan diester 24:0	n-17 to n-30	14	1299-1873	[M + Na]	y = 0.1401x + 185.90	R^2 = 0.9989	y= 7.4981x^0.5426	R^2 = 0.9997
POE Sorbitan diester 26:0	n-23 to n-28	6	1592-1813	[M + Na]	y = 0.1301x + 204.21	R^2 = 0.9965	y= 8.8529x^0.5206	R^2 = 0.9972
POE Sorbitan diester 28:0	n-24 to n-28	5	1664-1841	[M + Na]	y = 0.1429x + 183.19	R^2 = 0.9947	y= 5.7817x^0.5781	R^2 = 0.9952
POE Isosorbide	n-6 to n-19	14	433-1006	[M + Na]	y= 0.2014x + 99.68	R^2 = 0.9996	y= 5.5743x^0.5760	R^2 = 0.9986
POE Isosorbide	n-8 to n-21	14	533-1110	[M + K]	y= 0.1951x + 102.68	R^2 = 0.9993	y= 4.7561x^0.5987	R^2 = 0.9992
POE Isosorbide monoester 10:0	n-8 to n-17	10	675-1072	[M + Na]	y= 0.1777x + 131.88	R^2 = 0.9994	y= 7.6307x^0.5366	R^2 = 1.0000
POE Isosorbide monoester 12:0	n-5 to n-19	15	571-1188	[M + Na]	y= 0.1736x + 136.69	R^2 = 0.9994	y= 6.4105x^0.5611	R^2 = 0.9992
POE Isosorbide monoester 14:0	n-5 to n-19	15	571-1188	[M + Na]	y= 0.1656x + 146.02	R^2 = 0.9996	y= 8.2493x^0.5258	R^2 = 0.9985
POE Isosorbide monoester 18:1	n-10 to n-15	6	873-1094	[M + Na]	y= 0.1656x + 146.02	R^2 = 0.9991	y= 6.7080x^0.5575	R^2 = 0.9984
POE Isosorbide diester 22:0	n-14 to n-16	3	1121-1210	[M + Na]	y= 0.1393x + 184.03	R^2 = 0.9997	y= 12.6870x^0.4684	R^2 = 0.9994
POE Isosorbide diester 24:0	n-14 to n-16	3	1149-1238	[M + Na]	y= 0.1404x + 184.34	R^2 = 0.9992	y= 12.0970x^0.4758	R^2 = 0.9989
POE Isosorbide diester 26:0	n-15 to n-19	5	1221-1398	[M + Na]	y= 0.1320x + 196.99	R^2 = 0.9991	y=12.9400x^0.4671	R^2 = 0.9994
POE Isosorbide diester 28:0	n-12 to n-27	16	1117-1779	[M + Na]	y= 0.1462x + 179.85	R^2 = 0.9994	y=7.9632x^0.5356	R^2 = 0.9987
POE	n-6 to n-20	15	305-922	[M + Na]	y= 0.2158x + 92.28	R^2 = 0.9990	y=6.1871x^0.5615	R^2 = 0.9971
POE	n-8 to n-18	11	409-850	[M + K]	y= 0.219x + 87.92	R^2 = 0.9994	y=4.8990x^0.5953	R^2 = 0.9973
POE monoester 10:0	n-8 to n-18	11	547-988	[M + Na]	y= 0.1897x + 124.60	R^2 = 0.9988	y=7.8869x^0.5226	R^2 = 1.0000
POE monoester 12:0	n-6 to n-18	13	487-1016	[M + Na]	y= 0.1853x + 129.93	R^2 = 0.9992	y=9.4797x^0.5062	R^2 = 0.9995

PS-80

Series	Monomers	Points in Series	*m/z* Range	Adduct	Linear Fit Equation	R^2	Power Fit Equation	R^2
POE Sorbitan	n-21 to n-30	10	1111-1508	[M + Na]	y = 0.1592x + 140.34	R^2 = 0.9986	y= 4.8297x^0.5965	R^2 = 0.9990
POE Sorbitan	n-17 to n-28	12	951-1436	[M + K]	y = 0.1675x + 128.99	R^2 = 0.9992	y= 4.7864x^0.5973	R^2 = 0.9999
POE Sorbitan monoester 18:0	n-6 to n-18	13	717-1246	[M + Na]	y = 0.1699x + 144.62	R^2 = 0.9989	y= 8.1013x^0.5302	R^2 = 0.9999
POE Sorbitan monoester 18:1	n-10 to n-31	22	891-1817	[M + Na]	y = 0.1500x + 164.10	R^2 = 0.9988	y= 7.4825x^0.5406	R^2 = 0.9998
POE Sorbitan monoester 18:2	n-9 to n-19	11	845-1286	[M + Na]	y = 0.1619x + 150.07	R^2 = 0.9998	y= 7.9834x^0.5309	R^2 = 0.9998
POE Sorbitan diester 36:2	n-20 to n-26	7	1596-1861	[M + Na]	y = 0.1395x + 191.24	R^2 = 0.9965	y= 6.9582x^0.5539	R^2 = 0.9964
POE Isosorbide	n-7 to n-24	18	477-1226	[M + Na]	y= 0.1930x + 105.14	R^2 = 0.9983	y= 5.1209x^0.5886	R^2 = 0.9994
POE Isosorbide	n-9 to n-19	11	581-1022	[M + K]	y= 0.1985x + 99.77	R^2 = 0.9996	y= 4.4851x^0.5987	R^2 = 0.9993
POE Isosorbide monoester 14:0	n-10 to n-19	10	819-1216	[M + Na]	y= 0.1643x + 148.22	R^2 = 0.9998	y= 8.3208x^0.5252	R^2 = 0.9986
POE Isosorbide monoester 18:1	n-8 to n-18	11	785-1226	[M + Na]	y= 0.1645x + 148.70	R^2 = 0.9997	y= 8.5122x^0.5223	R^2 = 0.9998
POE	n-8 to n-20	13	393-922	[M + Na]	y= 0.2144x + 92.996	R^2 = 0.9988	y=5.2228x^0.5872	R^2 = 0.9989
POE	n-8 to n-20	13	409-938	[M + K]	y= 0.2145x + 90.23	R^2 = 0.9993	y=4.7837x^0.5989	R^2 = 0.9982
POE monoester 14:0	n-10 to n-17	8	691-1000	[M + Na]	y= 0.1758x + 140.17	R^2 = 0.9998	y=9.2372x^0.5110	R^2 = 0.9994
POE monoester 18:1	n-5 to n-18	14	525-1098	[M + Na]	y= 0.1768x + 139.99	R^2 = 0.9996	y=10.4138x^0.4941	R^2 = 0.9989
POE diester 34:2	n-10 to n-13	4	981-1114	[M + Na]	y= 0.1563x + 172.50	R^2 = 0.9991	y=11.3782x^0.4869	R^2 = 0.9995
POE diester 36:2	n-7 to n-12	6	877-1098	[M + Na]	y= 0.1659x + 163.86	R^2 = 0.9936	y=10.3561x^0.5011	R^2 = 0.9959

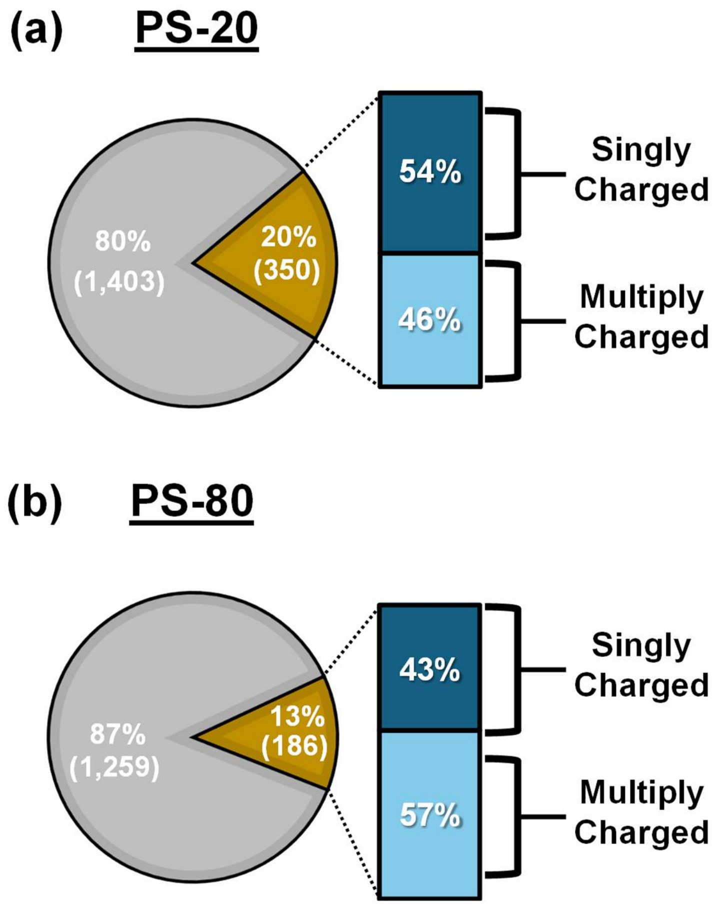

Figure S4. IM feature analysis of identified features (gold) and unidentified features (grey) using the ion mobility feature extraction (IMFE) tool within IM-MS Browser. Analysis of PS-20 **(a)** resulted in a preponderance of singly charged features relative to PS-80 **(b)**, suggesting the presence of larger PS species in the PS-80 mixture.
